# Development and proof-of-concept of a complex intervention to support appropriate imaging for musculoskeletal pain: the Betti programme

**DOI:** 10.1186/s43058-026-00949-4

**Published:** 2026-05-05

**Authors:** Nicole Lindner, Kristina Buch, Konrad Hierasimowicz, Reinhard Loose, Michael Walz, Karl-Friedrich Schüttler, Veronika van der Wardt, Annika Viniol

**Affiliations:** 1 Department of Primary Care, Philipps-Universität Marburg, Karl-von-Frisch-Str. 4, 35043 Marburg, Germany; 2 Department of Medical Physics, Paracelsus Medical School. Hospital Nürnberg, Prof.-Ernst-Nathan-Str. 1, 90419 Nürnberg, Germany; 3 Medical Institution for Quality Assurance in Radiology, Nuclear Medicine and Radiation Therapy, TÜV SÜD Life Service GmbH, Am Römerhof 15, 60486 Frankfurt, Germany; 4 Orthopaedicum Lich, Gottlieb-Daimler-Strasse 7A, 35423 Lich, Germany

**Keywords:** Musculoskeletal diseases, Implementation science, Primary health care, Program development, Quality improvement, Evidence-based practice, Health communication

## Abstract

**Background:**

Inappropriate diagnostic imaging for musculoskeletal pain is common and causes patient harm and unnecessary costs. Existing interventions frequently target single conditions and stakeholders and show limited integration into routine consultations. This study aimed to develop and test a complex intervention to de-implement low-value care by supporting appropriate imaging decisions for musculoskeletal pain in primary care.

**Methods:**

We developed *Betti* (“Better Imaging”) following the Medical Research Council framework (MRC) for complex interventions. The development was both theory- and evidence-based and included: specification and iterative refinement of a programme theory (logic model); a comprehensive literature review; qualitative interviews with patients; structured expert feedback; and a proof-of-concept test with general practitioners and patients. The Behaviour-Change-Wheel informed intervention components. We addressed early implementation considerations throughout the development process.

**Results:**

Findings from the literature review revealed a wide range of intervention approaches and components. Overall effectiveness was mixed. Reductions in imaging were reported more frequently in multicomponent interventions that included both physician-facing components and patient-facing materials. Qualitative interviews with people with musculoskeletal pain highlighted that expectations of primary care consultations are highly individual and shaped by context (e.g., prior experiences). Expert feedback emphasised communication and reassurance to support imaging decisions. Informed by these findings, we developed *Betti*, multicomponent intervention comprising: (1) a multimedia training module for general practitioners, (2) a clinical decision support system based on guideline recommendations across musculoskeletal pain, and (3) multimedia patient information materials. In the proof-of-concept test, patients and general practitioners perceived *Betti* as well structured and supportive. However, implementation challenges emerged: patients were not directed to the materials, indicating that *Betti* was not integrated into the consultation as intended. These findings led us to refine the programme theory, explicitly positioning consultation-integrated delivery and physician-mediated handover explicitly as essential for our program theory.

**Conclusions:**

*Betti* is a theory- and evidence-based, stakeholder-developed intervention. Early findings show high acceptability but underscore consultation-integrated delivery as critical. The study adds transferable implementation insights for de-implementing low-value imaging by specifying mechanisms, determinants, and strategy choices beyond tool use, informing further refinement and future feasibility and effectiveness–implementation evaluation.

**Supplementary Information:**

The online version contains supplementary material available at 10.1186/s43058-026-00949-4.

**Table:** 

Contributions to the literature
- Many imaging-reduction interventions target single conditions or stakeholders and fit poorly into routine care. This study demonstrated how to develop systematically a multicomponent intervention to address both clinical decision-making and patient communication across musculoskeletal conditions.
- Consultation-integrated delivery and physician handover of patient materials appear central mechanisms. This helps explain how digital interventions can be embedded in primary care to support de-implementation of low-value care.
- Digital decision support tools are frequently judged by frequency of their use. Effects may persist even as tool use declines, suggesting evaluations should distinguish tool use from learning effects and sustained practice change.

## Background

Musculoskeletal pain is a common problem, affecting most individuals at some stage in their lives. For example, up to 70% of people experience shoulder pain and 90% experience low back pain at some stage in their lives [1]. Specifically looking only at low back pain, it is the leading cause of activity limitation and work absence among people of all ages and socioeconomic backgrounds [2].

Despite relatively clear guidance on imaging for musculoskeletal pain, low-value care in form of inappropriate imaging remains common. Flaherty et al. found that more than 60% of lumbar spine MRIs and over 30% of MRIs conducted for shoulder and knee pain were inappropriate, accounting for more than 20% of annual imaging costs across these indications [3]. These alarming results of unnecessary imaging are supported by other studies, which consistently report high incidences of inappropriate imaging [4–6].

Low value tests are medical procedures, which provide no benefits or even cause harm [7]. In musculoskeletal pain, imaging becomes low value when it is used in situations in which guideline recommendations advise against routine imaging and when the result is unlikely to change clinical management. In such cases, inappropriate imaging causes harm in many ways, extending far beyond the financial impact on healthcare systems. In the United States alone, diagnostic imaging accounts for over $100 billion in annual healthcare spending [3]. More critically, inappropriate imaging can mislabel healthy people as diseased. Many “degenerative” findings reflect normal aging and are unrelated to pain – for example, nearly half of asymptomatic 80-year-olds show disc protrusion on spine imaging, and most asymptomatic knees show MRI abnormalities [8]. Mislabelling from inappropriate imaging can mislead general practitioners (GPs) and patients and cause harm: GPs may give unhelpful advice (from bed rest to unnecessary surgery) [9], and patients may develop fear of movement [1]. Furthermore, inappropriate imaging with ionizing radiation can increase long-term risks for patients.

GPs are often the first contact for musculoskeletal pain [10], and imaging is frequently discussed in consultations [11]. A synthesis of 68 qualitative studies found that patients and physicians often see imaging as validating “real” pain; physicians also order imaging to manage diagnostic/medico-legal risk and meet expectations. The authors therefore called for interventions that educate and support both physicians and patients and address individual patient needs [12]. However, many existing interventions aim to reduce unnecessary musculoskeletal imaging, but most target only a single condition (mainly low back pain), use isolated strategies rather than integrated components that support behaviour change and workflow integration, and focus on physicians with limited patient-facing elements [13–16].

Reducing low-value imaging is a de-implementation challenge: it requires not only introducing evidence-based practices but also actively discontinuing entrenched behaviours and addressing the determinants that sustain overuse (e.g., expectations, risk management, and communication) [17]. More broadly, de-implementation refers to discontinuing practices that are not effective, less effective or less cost-effective than an alternative, or are potentially harmful [18].

In summary, there is an unmet need for a complex, multicomponent intervention that targets the behaviour of both physicians and patients and is applicable across common musculoskeletal pain. To guide the systematic development of such interventions, the Behaviour-Change-Wheel (BCW) is widely used as a theory-informed framework that links behavioural determinants (capability, opportunity, motivation) to the selection of intervention functions and concrete behaviour change techniques [19]. The BCW has been applied to develop a range of theory-informed interventions, e.g. to reduce imaging overuse in low back pain [20] or to promote physical activity in chronic pain [21].

The aim of our study was to develop systematically a complex intervention by integrating theory, stakeholder involvement, and evidence. The resulting intervention itself aims to support GPs and patients in de-implementing low-value imaging by enabling informed, appropriate imaging decisions for musculoskeletal pain.

## Methods

We developed a complex intervention utilising a theory- and evidence-based approach. The development followed the framework of the Medical Research Council (MRC) and the guidance to develop complex interventions [22, 23]. Our multidisciplinary team (practising and academic GPs, psychologists, sociologist, IT specialist) supported by an expert group (six GPs, one orthopaedic specialist, two radiologists, three patient representatives, and an expert in plain-language editing) developed the intervention. All members were familiar with the German health system. The development process included a comprehensive internal review by the entire working group of the Department of Primary Care (Marburg, Germany) and external feedback (German Primary Care Conference and an online meeting with another research group). The entire development process was dynamic and iterative.

**Fig. 1:**
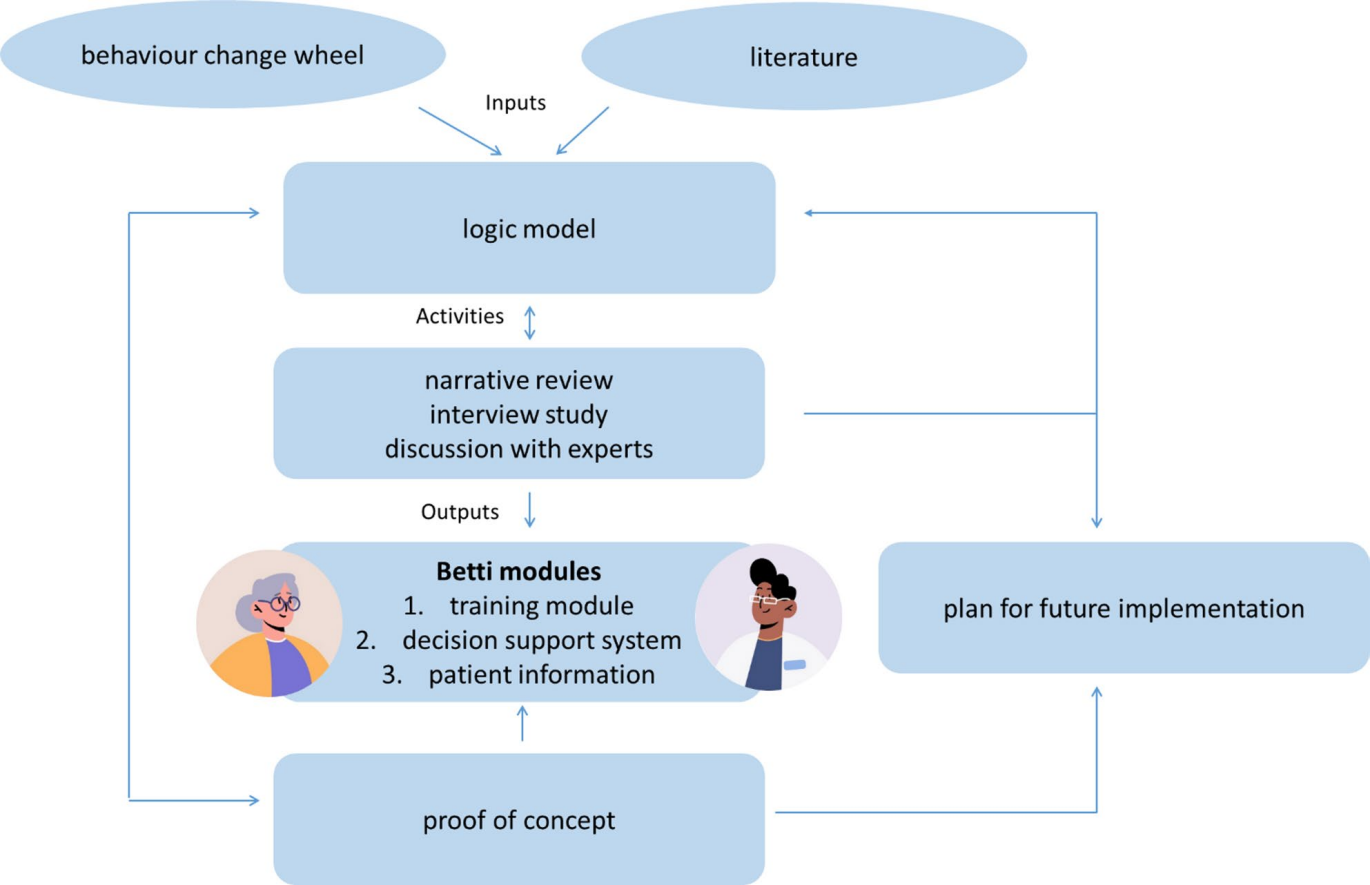
Overview of the development process. Inputs, activities and outputs refer the development of Betti and not to the program theory of Betti itself

Figure 1 outlines the intervention development process. Guided by the BCW [19] and informed by existing evidence on interventions to promote appropriate imaging (main inputs), we developed a programme theory in the form of a logic model [24]. Key development activities included a narrative review of interventions supporting appropriate imaging, interviews with people with musculoskeletal pain who had consulted their GP to explore experiences and expectations, and expert discussions on decision support for appropriate imaging. These activities informed the *Betti* intervention, which is presented in detail in Table 6. *Betti* was subsequently tested in a proof-of-concept study. In parallel, we developed an implementation plan based on findings from all stages.

### Development of the logic model

Firstly, we developed a programme theory in form of a logic model to guide development of the intervention [24]. We chose a logic model because it allows iterative refinement, is recommended by the MRC framework [23], and facilitates a shared understanding among the development team and experts. This programme theory was continually tested and refined throughout the development of the intervention.

The theoretical foundation of our programme theory was the BCW, with the COM-B system (Capability, Opportunity, Motivation, and Behaviour) as its centre [19]. We selected the BCW because it offers a comprehensive framework that integrates multiple theories, provides practical guidance for intervention design and the selection of behaviour change techniques, and is evidence-based. The BCW informed the translation of findings from the narrative review, interview study, expert discussions, and proof-of-concept study into intervention components. Potential intervention functions were identified from these data sources and discussed within the study team. During these discussions, the sources of behaviour relevant to imaging decisions were considered in relation to the COM-B model within the BCW framework and finally mapped to the intervention functions of the BCW. No formal APEASE (Acceptability, Practicability, Effectiveness, Affordability, Side effects/Safety, and Equity) assessment was undertaken [25]. Instead, intervention functions were selected based on their relevance to the identified sources of behaviour and their perceived feasibility and acceptability in primary care.

### Narrative review

We conducted a comprehensive literature search in form of a narrative review [26] in MEDLINE via PubMed to identify interventions aimed at improving the appropriateness of imaging for individuals with musculoskeletal pain. We considered all study designs, including quantitative, qualitative, multimethod, and mixed-methods, and all languages. We included all publications describing the development, evaluation, or testing of interventions intended to influence imaging use, imaging appropriateness, or imaging-related decision-making in people with musculoskeletal pain. Studies targeting healthcare professionals, patients, or both were eligible. We excluded studies on interventions that did not specifically address imaging, for example interventions focused solely on pain treatment. No search limits were applied. The search syntax is presented in supplement 1. Screening of titles and abstracts was carried out by a research assistant under the supervision of the first author NL, followed by full-text screening by NL and the research assistant of potentially relevant articles. Data, relevant for the development of *Betti*, were extracted by NL and subsequently reviewed by the entire study team to assess their relevance for the development of *Betti*. For each included publication, we extracted data on the first author and year of publication, the musculoskeletal pain region addressed, the target group, the study design and the way in which the intervention had been developed. We also extracted findings considered relevant to the development of *Betti*, including information on intervention components, delivery format and implementation considerations. The extracted data were synthesised narratively in a table because the included studies were heterogeneous in design, target population, intervention content, and outcomes. We did not conduct a formal quality appraisal because the aim of the review was not to provide a definitive assessment of effectiveness, but to inform intervention development by identifying a broad range of potentially relevant approaches, components, and implementation insights. The absence of a formal quality appraisal means that the strength of evidence underpinning individual findings cannot be judged properly. However, implications for the development of *Betti* were discussed within the study team.

Furthermore, for the development of the decision support system (module 2), we conducted a second literature search of national and international guidelines on imaging for musculoskeletal pain (e.g. National Institute for Health and Care Excellence guidelines, American College of Radiology Appropriateness Criteria, Guidance for Diagnostic Imaging). We searched systematically for each musculoskeletal region and the most common associated conditions. We included guidelines and guidance documents containing recommendations on imaging for musculoskeletal pain and excluded documents without imaging-related recommendations. The search was limited to documents published in English or German. We extracted data on the issuing organisation, musculoskeletal region or condition addressed, and the relevant imaging recommendations. These data were summarised in tabular form and synthesised narratively across body regions and conditions. We did not undertake a formal quality appraisal. However, wherever possible, we aimed to identify and include guidelines of the highest methodological standard. The extracted recommendations combined with clinical expertise from the multidisciplinary team and the expert group, formed the basis for a decision tree tailored to different types of musculoskeletal pain (*Betti* module 2: Decision support system for the use of imaging in musculoskeletal pain for GPs). Details on the search syntax can be found in supplement 1.

### Interview study

To understand the intervention context, we conducted semi-structured interviews with patients who had consulted their GP for musculoskeletal pain within the past year, building on Sharma et al.’s qualitative synthesis [12]. While Sharma et al. provided an international overview of imaging-related expectations, misconceptions, and decision-making processes, our interviews generated setting-specific insights to inform intervention design and adaptation. A semi-structured interview guide was developed by KB in dialogue with the research team and subsequently discussed with the patient advisory board. We did not conduct pilot interviews; however, the interview guide was refined continuously during the course of data collection. Initially, we aimed to recruit approximately 12–15 participants. Recruitment was stopped when the team judged that the collected interviews provided sufficient information power to address the study aim [27]. The transcripts were analysed thematically in six steps, following the method of Braun and Clarke and were analysed using a deductive– inductive approach; with interview questions supporting theme development (deductive) but participant answers allowing new themes to emerge (inductive) by the author KB [28]. Our approach was designed to identify the expectations regarding the consultation of patients with musculoskeletal pain and their lived experience in these healthcare interactions. Complete results from this analysis will be published elsewhere.

### Structured discussion with experts

**Fig. 2:**
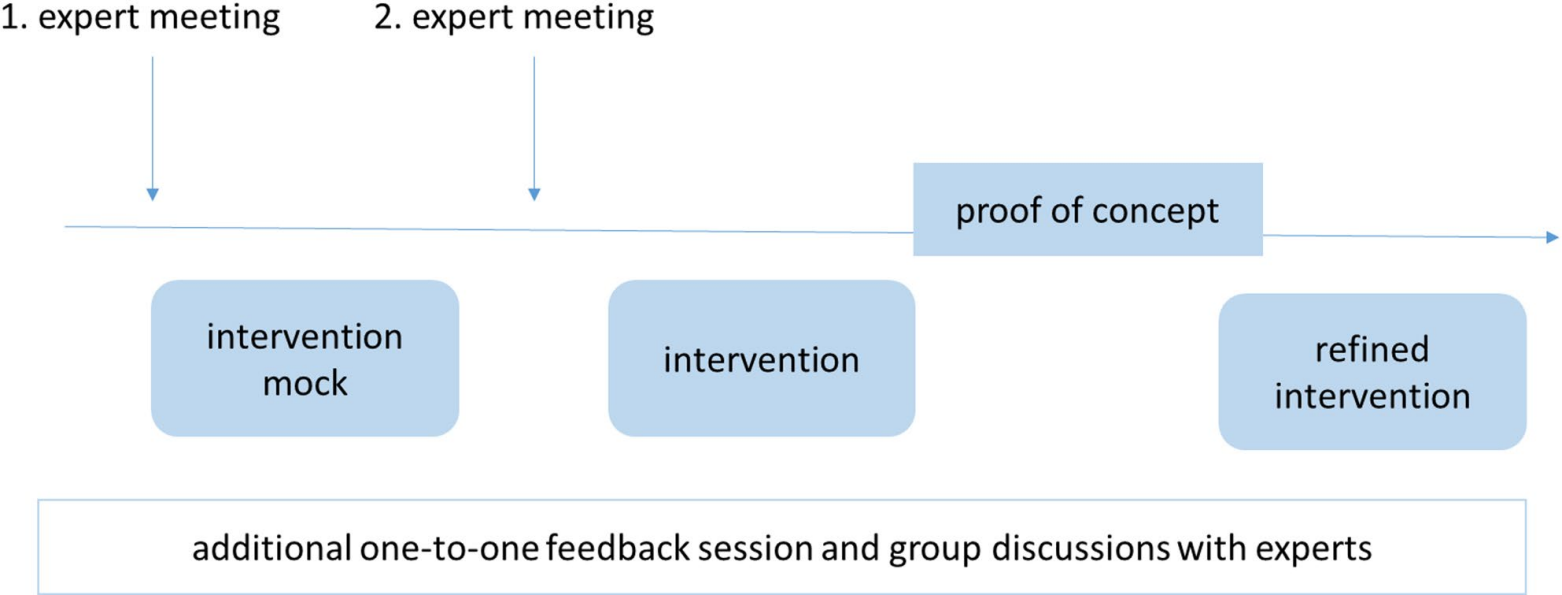
Overview of the involvement of experts

To incorporate diverse expertise, we established an expert group from the project planning stage and expanded it during the development to increase feedback. The final group compromised a mixed-clinician-patient panel, including six GPs, one orthopaedic specialist, two radiologists, three patient representatives, and an expert in plain-language editing. Views of experts were gathered via two expert group meetings (not all experts attended every meeting) to discuss broader topics and promote interactive discussion. Additionally, we conducted several one-to-one feedback sessions as well as smaller group discussion for specific points (in person, via telephone, or via video conference). An overview of the involvement of experts can be found in Fig. 2. In the first meeting, we discussed challenging clinical situations related to decisions on musculoskeletal pain, as well as the type and format of information that could best support GPs and patients in making imaging decisions. In the second meeting, an intervention mock was presented to the experts. The experts reviewed the content and application format. In the expert group meetings, creative activities such as role play were used to simulate real consultation scenarios. Specifically, pairs of experts worked through simulated clinical cases and used the decision support system in real time to guide management decisions. As the group intentionally combined clinicians and patient representatives, we acknowledge the potential for power imbalance within the discussion. To mitigate this, we supplemented the mixed group meetings with one-to-one feedback sessions and smaller group discussions, providing additional space in a less hierarchical system. In the group discussions, we also deliberately prioritised the perspectives of patient representatives, for example by inviting them to speak first and by specifically asking them for their views.

### Proof-of-concept study

After the second expert meeting and refinement of *Betti*, we performed a proof-of-concept study with two GPs and four patients in Hesse (Germany). Given the early developmental stage of the intervention, we used a small pragmatic sample to obtain initial feedback on usability, acceptability, and integration into routine consultations, rather than to evaluate effectiveness or achieve thematic saturation. GPs were recruited via our research practice network. Participating GPs then recruited patients from their practices who had taken part in a consultation in which Betti was used. All participants provided written informed consent prior to participation. The first author NL presented *Betti* to the GPs and they further familiarised themselves with the different parts on their own. Subsequently, each GP applied *Betti* in at least two consultations addressing different musculoskeletal pain. After these consultations, NL conducted semi-structured retrospective interviews with the GPs to explore usability, added value, and difficulties in using *Betti*. During these interviews, *Betti* was opened again and discussed jointly as a guided walkthrough to support reflection on prior use in practice. An outline of the interview guide can be found in supplement 2. The interviews were pseudonymised, transcribed verbatim, and thematically analysed (Braun and Clarke) by NL [28]. All four patients in the imaging consultations were invited by their GP for an interview; after written consent, NL conducted the interviews. Data collection and analysis followed the same qualitative research procedures as described above.

### Plan for future implementation

In line with the updated MRC framework, we considered future implementation in primary care from the outset [23]. Implementation was discussed in internal team meetings and expert consultations and informed by exchange within a broader academic network on implementing complex interventions in primary care. Evidence from the literature further informed and refined the implementation strategy. Anticipated implementation outcomes were considered in relation to the key domains proposed by Proctor et al. [29, 30], and strategies were specified using the Expert Recommendations for Implementing Change (ERIC) taxonomy of implementation strategies [31]. For the development we mainly focused early concepts on implementation of *Betti* and less specifically on concepts regarding de-implementation of inappropriate imaging [32].

### Design of the modules of the Betti intervention

The first draft of Betti was developed through an iterative process within the multidisciplinary core study team. After individually reviewing the available evidence, team members generated initial ideas and draft materials for the different components of the intervention. NL prepared the first draft of the decision support system, whereas KB prepared the first drafts of the training module and patient information materials. These draft components were first discussed within the study group and were then presented to the expert group at an early stage of development. No formal consensus method was used; instead, the draft was developed through iterative multidisciplinary team discussion and agreement. We developed three different modules as part of the *Betti* intervention. The intervention is described in detail in Table 6. The modules can be accessed via a homepage (www.entscheidung-bildgebung.de). Details on the technical specifications of the decision support system can be found in supplement 3.

### General methodological aspects

The study was conducted in accordance with the Declaration of Helsinki and received ethical approval from the ethics committee of the University of Marburg (AZ 25–232 BO) [33]. We used ChatGPT (version 5.2) for text editing (all content and interpretations remain the responsibility of the authors).

## Results

### Development of the logic model

**Fig. 3:**
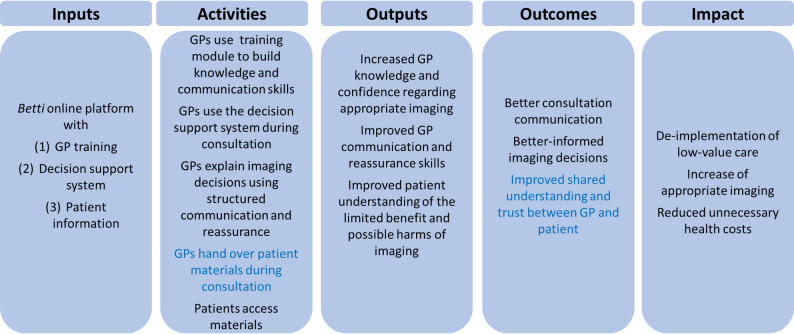
Logic model of the Betti-intervention. Elements that became especially salient during development are marked in blue

Firstly, we developed a programme theory in the form of a logic model to describe the mechanisms through which the intervention is expected to influence imaging decisions in primary care (illustrated in Fig. 3). This model guided the iterative development of *Betti* and was continuously adapted throughout the process.

GPs are expected to use the training module to increase knowledge of appropriate imaging and to further develop communication skills. During consultations, GPs are expected to use the decision support system to support imaging decisions, explain these decisions using structured communication, provide reassurance, and hand over the patient materials. Patients are then expected to access the patient information materials. These activities are expected to increase GP knowledge and confidence, improve communication and reassurance, and enhance patient understanding of the limited benefit and potential harms of imaging. Together, these outputs are intended to improve consultation communication, support better-informed imaging decisions, and strengthen shared understanding and trust between GPs and patients. This in turn is intended to contribute to the de-implementation of low-value imaging, promote more appropriate imaging, and reduce unnecessary healthcare costs.

Across the different stages of development, GP-mediated handover of patient materials and improved shared understanding and trust between GPs and patients became apparent as especially central elements of the *Betti *programme theory.

### From theory to components: BCW-guided operationalisation

Application of the BCW indicated that inappropriate imaging is influenced by determinants at the level of both GPs and patients. For GPs, relevant determinants included limited knowledge of guideline-consistent imaging, uncertainty in complex consultations, perceived pressure from patient expectations, and challenges in explaining why imaging is not indicated. These determinants were considered to relate mainly to psychological capability, social opportunity, and reflective motivation. For patients, key determinants included limited understanding of the role and limitations of imaging, anxiety, and expectations shaped by previous experiences, corresponding mainly to psychological capability and reflective motivation.

**Fig. 4:**
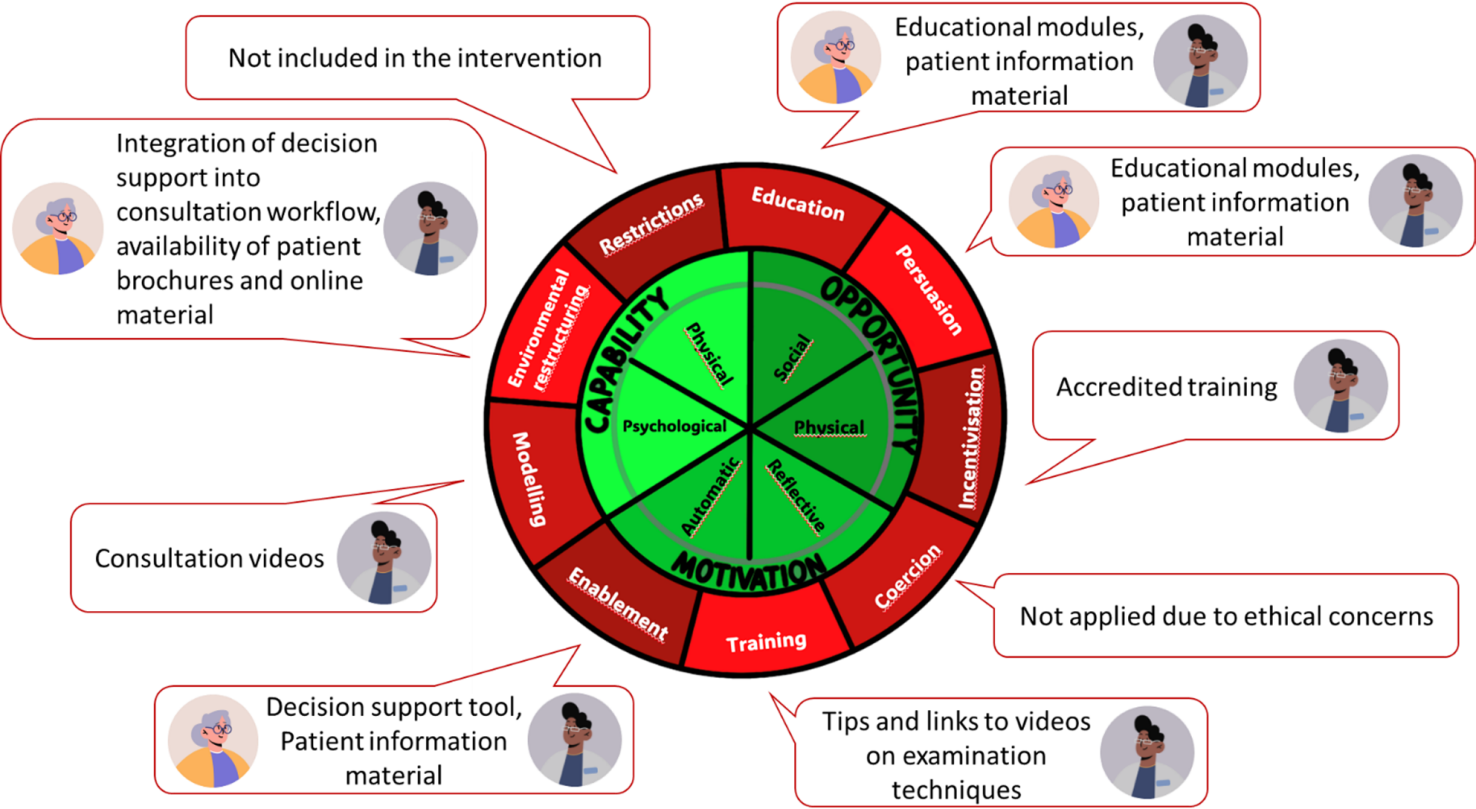
Mapping of Betti programme parts to targeted dimensions. Behaviour Change Wheel (BCW) according to Michie et al. [19] (yellow patient symbol=patient-targeted component, grey doctor symbol= physician-targeted component)

Based on the determinants our intervention targets multiple dimensions of the BCW to support both physicians and patients. For physicians, the intervention employs the dimension ‘Education’ to enhance their understanding of appropriate imaging for musculoskeletal pain, a decision support system ‘enables’ them to make informed choices during consultations, and tips and links to examination techniques help them to ‘train’ their skills to determine when imaging is appropriate. Additionally, videos showcasing typical consultations serve as ‘modelling’ tools. For patients, the intervention provides ‘education’ with patient information materials on the appropriate use of imaging and the associated risk. It also ‘enables’ them to actively participate in the decision-making process. We deliberately chose not to employ the dimension of ‘coercion’, as we did not consider it ethically justifiable; instead, our aim is to contribute to informed decision-making and to support, rather than pressure, physicians and patients. Figure 4 maps the *Betti* components to targeted dimensions.

### Results of the narrative review on existing interventions

We conducted the search in January 2025 and identified 4,174 articles. After screening titles and abstracts, we assessed 146 full-text articles for eligibility. Ultimately, we included 32 publications describing interventions aimed at improving the appropriateness of diagnostic imaging in individuals with musculoskeletal pain. The main reasons for exclusion at the title and abstract screening stage were that publications did not report an intervention, were not concerned with imaging-related decision-making, or did not address musculoskeletal pain. At the full-text stage, the main reason for exclusion was that publications did not describe an intervention focused on imaging-related decision-making in musculoskeletal pain, for example because they addressed treatment of musculoskeletal pain rather than imaging.

Key results of the narrative synthesis of the 32 included publications are presented in Table 1. The insights from this review directly informed the development of *Betti* and led to adaptions of the logic model. Examples are also presented in Table 1 and Supplement 4 provides an overview of the key findings from literature review and details how these insights informed the development and content of *Betti*.

**Table 1:** Examples how results of the narrative review informed the design of Betti

Key results of the narrative review	
- Most interventions targeted healthcare professionals (*n* = 16) [15, 34–36], while fewer targeted patients (*n* = 6) [14, 37, 38] or both patients and professionals (*n* = 10) [39, 40]. - Nearly all interventions focused on back pain (*n* = 29) [13, 37] —predominantly low back pain- and only a small minority addressed other musculoskeletal presentations (e.g., knee or hip pain) [36, 41]. None of the identified interventions aimed to address a broad range of musculoskeletal pain presentations within one integrated intervention. - Interventions were mainly intended for emergency departments [15, 42] and primary care [13, 43]. - Across studies, intervention components clustered into recurring strategies: (i) decision support systems and ordering requirements (e.g., electronic prompts, mandatory indication checks, structured documentation aids), frequently combined with audit-and-feedback or peer comparison [13, 44]; (ii) physician education and communication training (workshops, e-learning, simulated/standardised patient encounters) [34, 43]; and (iii) patient-facing education and communication tools (brochures, videos, and web portals) [37, 41]. - Overall effectiveness was mixed. Reductions in imaging were reported more consistently in multicomponent interventions that combined physician with patient-facing materials and when interventions were embedded in routine workflows and reinforced by feedback mechanisms [39, 45].	

Specific result	Key adaptions
More promising approaches combined physician- and patient-facing components	*Betti* was designed as a multicomponent programme
Evidence from studies on a web portal for back pain (“tala-med”) shaped format and delivery	While the layout of the web portal informed *Betti’s* structure, its evaluation highlighted that patient-facing tools only reach patients effectively—and do not risk impairing perceived communication quality—when GPs actively introduce and use them during the consultation [40, 46].
Behavioural-economics “nudging” strategies	Behavioural-economics “nudging” strategies were considered, but given ethical concerns raised in the evidence base, they were not adopted as an mechanism; instead, only low-coercion elements (e.g., clear cues to action guiding patients towards appropriate next steps and alternatives) were incorporated [38].
Checklist-style diagnostic algorithms	While checklist-style diagnostic algorithms have been proposed [47], this approach was discussed in the second expert meeting and rejected as insufficiently feasible across heterogeneous musculoskeletal presentations; accordingly, *Betti* integrates concise examination tips within the decision support system.

### Results of the interview study

We interviewed 18 people with musculoskeletal pain in different anatomical regions from March to October 2025. Key results included that people with musculoskeletal pain want an explanation for their pain, want to be heard, seen and taken seriously, and that expectations regarding next steps and further treatment planning (e.g. whether a referral for imaging is expected) are individual and influenced by individual context (e.g. previous experiences). The complete results of the interview study will be published elsewhere an overview of results and key adaptions is presented in Table 2.

**Table 2:** Summary of the results of the interview and key adaptions to Betti and the logic model

Theme	Key finding	Resulting modifications
Overall understanding and context	Deeper understanding of the intervention setting	Incorporated into the overall intervention development
Individuality of expectations	Expectations regarding next steps and further treatment planning are individual	Refinements to communication training content (Module 2)

### Results of the discussion with experts

Input from the multidisciplinary expert panel was crucial in shaping *Betti*. Table 3 provides a summary of the feedback of the multidisciplinary expert panel and the resulting key adaptations to *Betti* and to the logic model. Across the two meetings and additional feedback sessions, the expert panel identified a strong need to support both patients and physicians in deciding for or against imaging in musculoskeletal pain. Their feedback informed iterative revisions of all three modules, including streamlining and standardising the decision support system and translating communication priorities into GP training and patient information, alongside initial recommendations for implementation and dissemination. A central emphasis was that imaging does not necessarily change management and should be communicated in ways that build shared understanding and reassurance. Together with findings from the narrative review and proof-of-concept test, this led to refinement of the logic model, positioning consultation-based shared understanding and reassurance as a key mechanism.

**Table 3:** Summary of multidisciplinary expert panel input across two meetings and one-to-one feedback sessions/ smaller group discussions, and the resulting key adaptations to Betti and the logic model

Topic	Key insights/ recommendations	Resulting modifications
**Expert meeting 1**		
Overarching need	Strong need for structured support for imaging decisions in musculoskeletal pain (patients and physicians)	Informed overall multi-module design (Modules 1–3).
Challenging scenarios	Imaging decisions are difficult when patients are insistent, pain site is atypical, clinical assessment diverges from expectations, or distress is high. (physicians)	Prioritised communication training content (Module 2)
Patient needs in challenging scenarios	Patients wanted an explanation for pain or a time-bound plan, plus transparent, convincing reassurance that signals being taken seriously. (patients)	Shaped consultation communication strategies (Module 1) patient messaging (Module 3) Updated logic model: shared understanding and reassurance in consultation is central
Core messages about imaging	Make explicit that imaging often does not change management and findings correlate imperfectly with function/pain (patients and physicians)	Integrated into GP training (Module 1) and patient materials (Module 3) Updated logic model: shared understanding and reassurance in consultation is central
Supports for physicians	Prioritised a point-of-care decision support system; body-region–specific guidance with consistent structure, typical presentations, examination tips; allow clinical judgement to diverge from tool suggestions (physicians)	Operationalised in Decision support system (Module 2)
Support for patients	Patient materials should include: explanation/plan; imaging limitations; downsides; use numbers and brief examples to convey likelihoods; provide multiple formats (brochure, video, podcast) (patients)	Informed multi-media patient information material (Module 3)
**Expert meeting 2**		
Review of GP training material (module 1)	Translated priorities into teachable communication skills; generated concrete phrasing for: explaining non-indication, decoupling imaging findings from pain/function, reassurance, negotiating insistent requests; recommended short modular videos to address time constraints. (patients and physicians)	Refined training content and format (Module 1)
Review of decision support system for patients (module 2)	Judged helpful/ accurate/ practicable; recommended simplified structure (fewer clicks), condition-specific referral template, copy-ready documentation text for normal findings; discussed more radical refinements diagnostic algorithms/ checklists, rejected due to limited evidence and scope (patients and physicians)	Refined decision support system (module 2), e.g. simplification/ standardisation of structure Integration of copy-ready texts for documentation and referral
Review of patient information material (module 3)	Overall positive feedback; refinements to wording and presentation (brochure/web); discussed an ultra-easy version (balanced against risk of information loss); podcast well received (suggested short condition-specific episodes); animated video content appropriate but execution suboptimal (slower pacing, calmer visuals, narration adjustments) (patients and physicians)	Refined patient information material (module 3)
Ideas on future implementation	Recommend CME-accredited training; involve additional specialties (radiology, orthopaedics) to support uptake; dissemination via national journals targeting physicians and patients (patients and physicians)	Informed implementation planning and dissemination strategy
**One-to-one-feedback sessions/ smaller group discussions**		
Review of decision support system for patients (module 2)	Was considered easy to use and recommendations were perceived as accurate. Suggestion to integrate additional pain conditions, discussed more radical checklist-style redesign, but rejected due to limited diagnostic value of signs	Integration of more conditions into the decision support system (module 2)
Review of patient information material (module 3)	Refinements to wording and presentation (brochure/web) for better understanding	Refined patient information material (module 3)

Betti consists of three modules: Module 1: multimedia communication training for GPs, Module 2: Decision support system for the use of imaging in musculoskeletal pain for GPs, Module 3: Multimedia patient information on imaging in musculoskeletal pain. CME: Continuing Medical Education

### Results of the proof-of-concept test

We included two GPs (one woman and one man, aged 38 and 39 years, with 8–9 years of experience in general practice, working in rural practices, both involved in medical teaching) and four patients (male and between 41 and 64 years of age) in the proof-of-concept test. All interview partners were White and had a German background. Interviews were held in November and December 2025. GP interviews were conducted in person and patient interviews via videoconference.

Overall, GPs and patients considered *Betti* as relevant, well structured, and closely aligned with routine primary care practice. The intervention was perceived as supportive, with physicians reporting that *Betti* largely confirmed their existing clinical approach, while patients felt taken seriously during the consultation. Both groups highlighted that trust and the interaction during the consultation itself played a central role in shaping decisions about imaging.

A key implementation challenge emerged: none of the patients had viewed the provided materials before their interview. Patients reported not having been directed to the materials by their GP or not having sufficient time to review them. This highlighted the need for a stronger connection between physician- and patient-facing components. Suggested improvements included integrating patient information directly at the end of the decision support system or offering QR codes during the consultation. This finding is particularly important because patients viewed the consultation as pivotal for imaging decisions, while GPs reported that repeated decision support use led them to internalise the process and stop using it in consultations, potentially reducing prompts to direct patients to the accompanying materials. In particular, findings on the central role of the consultation and the limited handover of patient materials led us not only to refine *Betti* but also to revise the logic model. We strengthened the emphasis on consultation-integrated delivery of the patient information and on improved consultation communication to foster shared understanding and reassurance between GPs and patients.

Physicians noted that consultations using *Betti* required additional time and viewed this as a barrier. They also expressed a wish for more individualised information tailored to specific pain presentations, such as simple joint-level visualisations and condition-specific explanations for why imaging is or is not appropriate. This was echoed by patients, who requested illustrations (e.g., “what is a herniated disc vs. lumbago?”). Furthermore, patients and physicians valued additional content beyond imaging, including brief exercises and information on red flags, ideally provided as easily accessible links or modules that can be shared directly with patients.

The communication guidance within *Betti* was perceived as the least helpful component for the experienced GPs in the proof-of-concept test. Nonetheless, they considered it potentially useful for less experienced physicians. Physicians emphasised that decisions around imaging are often driven by patient anxiety and concerns about medico-legal consequences. They felt that *Betti* could support these situations by offering structured explanations and clear wording for both patient communication and documentation.

Table 4 provides an overview of key findings of the proof-of-concept test with illustrative quotes.

**Table 4:** Overview of key findings from the proof-of-concept test and resulting modifications to the Betti intervention

Theme	Key findings (GPs)	Key findings (patients)	Resulting modifications
Positive overall assessment	*Betti* confirmed existing clinical approach; perceived as relevant and practice-oriented	General positive impression	none
	*“So personally*,* what I noticed is*,* well*,* I think it’s very*,* very good.”* (physician_A01)	*“I think the way the website is designed is just fine. Everything has a nice clear layout*,* and it’s easily understandable.”* (patient_P31)	
	*“I actually felt more reassured than I thought Oh*,* there are things in there that weren’t clear to me. Basically*,* I think I’ve been already doing it this way*,* which is good*,* but I don’t think many of my colleagues do it this way.”* (physician_A01)		
Access / interface between GP tool and patient materials	Limited usability due to missing “interface” to patient material	Materials not accessed	Strengthened linking between GP and patient components (QR-code, options to provide link)
	*“I don’t find it particularly helpful during consultations with patients because I can’t really show them anything properly while they are there.”* (physician_03)	*“I haven’t received anything. No.”* (patient_P31)	
Time burden in consultation	Additional time during consultation seen as barrier	none	none
	*“After doing it twice*,* I knew I had to take my time. I knew that the others would ultimately have to wait. On that day*,* I simply accepted that. But when it’s part of your daily work*,* you view many things quite differently.*“ (physican_A01)		
Individualisation of content	Wish for condition-specific information for patients	Wish for condition-specific information for patients	Added specific individualised information at the end of the decision support system
	*“It would probably help to move toward individualised information*,* patient information*.” (physician_A03)	*“But what exactly happens there [back pain] and how it differs from something more dramatic like a slipped disc probably looks completely different. Yes*,* if that could be shown in images or so*,* yes*,* yes*,* I think that would make it easier to understand.”* (patient_P11)	
Content beyond imaging	Wish for information beyond imaging	Wish for information beyond imaging	Not incorporated, due to *Bettis* focus on imaging
	*“So*,* at the moment*,* it’s a bit of a patchwork. I’ve picked out videos (…) that I send out*,* and sometimes I send out this and sometimes I send out that. And if I had everything from a single source and didn’t even have to make the videos myself*,* but just had to say*,* “You can find it here and here*,*” then I think that would be an added value*,* because it is and seems more individual for the patient.”* (physican_A03)	*“If you can somehow*,* well*,* in my case it was first of all rest*,* but at the same time exercise*,* doing everything that doesn’t cause pain*,* promoting blood circulation. (…) I think that’s also an important point. So*,* what can the patient do themselves? I think that should definitely be part of the visualization and/or this brochure.”* (patient_P32)	
Addressing fear and medico-legal concerns	Imaging sometimes driven by fear and legal reassurance needs; *Betti* can provide security	Patients are sometimes driven by fear, *Betti* can provide trust/security	Added a “copy-ready” sentence for use both in the medical record and in patient communication, clearly explaining why imaging is not indicated following *Betti*-supported assessment
	*“Yes*,* and also to feel a bit more secure*,* because not doing something can be very difficult at first.”* (physican_01)	*“Yes*,* well*,* if you. Well*,* if you have the trust*,* yes*,* that’s how it’s been explained to me and that’s what I believe now. And I don’t have to do that [imaging]. Yes*,* if you*,* if you believe that yourself*,* yes.”* (patient_P11)	
Communication module (GP training)	Considered least helpful for highly experienced GPs, potentially useful for less experienced colleagues	Patients were satisfied with communication	Planned focus on GP trainees in future implementation
	*“Yes*,* that’s all right and good*,* but when I look at everything*,* I actually find it the least helpful for me personally (…) It might have been really helpful to me four years ago.”* (physician_A01)	*“She [GP] had put in a lot of work.”* (patient_P11)	
Study procedures/ recruitment	Recruitment took several weeks; procedures perceived as cumbersome and disruptive *“That has*,* of course*,* changed the whole interaction between doctor and patient*,* because it became so bureaucratic*,* and I also noticed that I became a little uncertain*,* because I hoped that I had done everything right and given the right piece of paper to the right person.”* (physician_A01)	No negative comments on study procedures	simplification of procedures where possible
Candidate outcomes for a future RCT	Less imaging (physician_A03, physician_A01) consultation satisfaction (physician_A03, physician_A01) Self-efficacy (physician_A01)	Less imaging (patient_P11, patient_P32) Trust (patient_P11) Comparison Recommendation of *Betti* and what was actually done (patient_P12) General course of the disease (patient_P32) Self-efficacy (patient_P31)	Outcome set of the feasibility trial expanded

### Plan for future implementation

From the beginning, we considered future implementation of *Betti* as a key component of its development. Details on the results including mapping in terms of Proctor’s implementation outcomes [29, 30] and ERIC taxonomy of implementation strategies [31] can be found in supplement 6.

We mapped early considerations from the expert feedback and the proof-of-concept test to Proctor’s implementation outcomes. Feedback showed high acceptability as *Betti* was perceived as relevant, well structured, and supportive. However, adoption seemed to differ across components as no patients accessed the patient materials. *Betti* was perceived as practice-oriented (Appropriateness), but more condition-specific information was requested. Feasibility concerns mainly related to additional time requirements and the missing interface linking physician tools to patient-facing materials. Fidelity seemed to depend on consultation-integrated delivery and an effective GP–patient handover to the materials, which was not achieved in the proof-of-concept test. Implementation costs are likely to arise primarily from additional physician time, while potential system-level savings may accrue, if *Betti* reduces cost-intensive low-value imaging. Regarding planned penetration we explicitly considered the components of an implementation strategy. and organised them according to the ERIC taxonomy of implementation strategies (e.g. CME-accredited sessions, targeting GP trainees) (details in supplement 6).

### Design of the *Betti* intervention

The design of *Betti* was informed by the findings of the narrative review, interview study, expert discussions, and proof-of-concept study. Table 5 summarises the key findings from these development components and the resulting adaptations to *Betti*.

**Table 5:** Cross-source overview of how key findings from each development informed the Betti intervention and the programe theory

Development component	Key finding	Resulting adaption to Betti	Implication for programe theory
Narrative Review	Multicomponent interventions combining physician- and patient-facing elements appeared more promising than single-component approaches	*Betti* was designed as a multicomponent intervention with GP training, decision support, and patient materials	Supported the assumption that reducing low-value imaging requires coordinated support for both GPs and patients
	Existing interventions mainly focused on low back pain rather than a broader musculoskeletal spectrum	*Betti* was developed across common musculoskeletal pain presentations rather than one body region only	
Interview study	Expectations regarding imaging and next steps were highly individual and shaped by prior experiences	Communication guidance was refined to support more individualised discussions during consultations	Highlighted the need for flexible, patient-centred communication
Expert disussions	Strong need for structured support for imaging decisions in musculoskeletal pain	Confirmed the overall design of *Betti*	Supported the relevance of the intervention concept Strengthened patient-facing mechanisms of reassurance and understanding
	GPs needed point-of-care, body-region-specific decision support with a consistent structure	Module 2 was refined as a structured decision support system with concise examination tips and region-specific guidance	
	Patients needed clear explanation, reassurance, and accessible materials in different formats	Module 3 was developed as multimedia patient information (brochure, web, video, podcast)	
	Imaging should be explained as often not changing management and findings not always matching pain/function	Core communication messages were integrated into GP training and patient materials	
Proof-of-concept study	Patients often did not access the patient materials because they were not actively directed to them	Linking between GP-facing and patient-facing components was strengthened, e.g. clearer handover pathways / QR code / direct links	Placed further emphasis on the use of Betti during consultations and strengthened physician-mediated handover as key mechanism
	Users wanted more tailored, condition-specific information	Additional condition-specific information was added where feasible, especially at the end of the decision support pathway	

We have designed *Betti* consisting of the three modules, which can be accessed via a homepage (www.entscheidung-bildgebung.de). In Table 6 we present *Betti* in the TiDieR-format (template for intervention description and replication) and in supplement 5 you can find an impression of *Betti* [48].

**Table 6:** Description of the intervention in the TiDieR-format. GP: general practitioner, MSK: musculoskeletal

**Brief name** Betti (“Better Imaging”) an intervention to support appropriate imaging decisions in musculoskeletal pain (MSK) in primary care (de-implementation of low-value care)
**Why** inappropriate imaging for MSK pain is common, costly and harmful Betti aims to reduce unnecessary imaging by supporting patient-centred-communication, increasing knowledge and confidence of general practitioners (GPs) and patients Betti is grounded on the Behaviour Change Wheel and a logic model theory
**What – Materials** Betti compromises several key components, which can be accessed via an online platform: 1. GP training materials, which provide background information on appropriate imaging for MSK pain and strategies for patient centred communication, these include Example consultations in video format Online information portal 2. Decision support system for GPs, based on international and national guidelines, structured as a decision tree adapted to common MSK conditions (e.g. shoulder, knee, back pain) 3. Multimedia patient education materials, which explain the appropriate use of imaging and its potential risk, these include: educational video audio podcast online information portal printed brochure
**What – Procedures** *Betti* was designed to address common musculoskeletal pain presentations across body regions using a shared intervention structure, while the decision support system included body-region-specific pathways and recommendations where clinically relevant. *Betti* is designed for flexible use by both patients and GPs, and can be integrated in routine care primary care in the following ways: **For patients**: Access the intervention materials independently or via a link provided by the GP through the *Betti* homepage Read the printed brochure available in GP surgeries Listen to the podcast Receive recommendations and explanations of *Betti* by their GP during the consultation **For GPs**: Use the training material to prepare for imaging-related consultations Apply the decision support system during consultations to support the decision-making process Guide patients to the educational material
**Who provided** GPs apply the decision support system during consultation and independently prepare using the training materials Patients access educational materials independently
**How** *Betti* is delivered through a combination of face-to-face and digital approaches: Consultation-based delivery: GPs apply the decision support system directly during the consultations to support decision making Self-guided GP preparation: GPs prepare independently through online training materials and example consultation videos Digital access for patients to multimedia educational content Printed materials available in GP surgeries Indirect delivery through GP recommendations
**Where** Online platform, printed brochures
**When and how much** Patients and GPs can access *Betti* flexibly multiple times
**Tailoring** GPs can choose which training material (video, text) they want to use GPs are encouraged to personalise communication strategies GPs can tailor the decision support system based on the type of MSK pain and clinical presentation Patients can choose which educational material (e.g. podcast, video, printed brochure, online material) they want to use
**Modifications** *Betti* was modified iteratively during the development based on the literature search, interview study, expert feedback and proof-of-concept test
**How well planned** Fidelity and adherence were assessed qualitatively through post-consultation semi-structured interviews with GPs and patients, including a think-aloud walkthrough of *Betti* to determine whether core components were used as intended. Results were used to refine the program theory and the intervention to strengthen consultation integration. Fidelity was supported by a standardised introduction to *Betti* and self-learning material.
**How well actual** Both GPs of the proof-of-concept test used *Betti* in at least two consultations for different musculoskeletal pain presentations and reviewed its components in the interviews. However, patient materials were rarely accessed, indicating incomplete physician handover and limited consultation-integrated delivery; GPs also reported relying less on the decision support system after repeated use.

## Discussion

We developed and tested *Betti*, a multicomponent, theory-informed intervention to de-implement low-value primary care via reducing potentially harmful overuse of imaging for musculoskeletal pain. Combining evidence, qualitative insights, and expert input, the intervention targets GPs and patients. *Betti* consists of three modules: (1) a multimedia training module for GPs providing background knowledge and communication strategies; (2) a decision support system offering body region-specific, guideline-based recommendations for imaging; and (3) patient-facing educational materials available in multiple formats, including a brochure, an online information portal, a video, and a podcast. The proof-of-concept test indicated good relevance but also a need for better integration into routine care.

### Comprehensiveness of Betti: advantages and challenges

To the best of our knowledge, *Betti* represents the most comprehensive intervention to date that simultaneously targets patients and physicians and addresses multiple body regions of musculoskeletal pain within a single, integrated programme. Existing approaches typically focus on either a single stakeholder group—most commonly physicians—or restrict their scope to one anatomical region, most often low back pain [49]. By contrast, *Betti* combines physician training, a decision support system, and multimedia patient education across several common pain presentations. This breadth is intended to reflect the realities of primary care, where musculoskeletal complaints are heterogeneous and where effective reduction of unnecessary imaging requires coordinated support for physicians and patients [12].

This comprehensiveness may offer practical advantages, including broader applicability across common musculoskeletal presentations and greater scalability for routine primary care. Systematic review evidence suggests that multicomponent interventions are more frequently associated with reductions in low-value imaging – and low-value medical testing in general - than single-component approaches [7, 50]. At the same time, a broader scope increases complexity and implementation burden and may reduce usability if components are perceived as too extensive or insufficiently tailored [51–53]. Reason for this might be that covering a wide range of conditions often necessitates longer or more generic content, which may be experienced as “one-size-fits-all” and insufficiently adapted to specific patient presentations, workflows, or local resources.

Furthermore, a broader scope can also complicate maintenance (e.g., keeping guidance current across regions/conditions) and evaluation of the intervention (efficacy, context interaction), as effects may differ by body region and setting [23, 54].

### Key lessons learned during intervention development

Beyond *Betti*, this study contributes transferable knowledge on de-implementing low-value imaging by specifying and practice-testing consultation-integrated mechanisms.

Especially important for our development process was the MRC framework’s emphasis on iteration [23]. Insights generated through successive development activities (e.g., expert input and proof-of-concept testing) repeatedly prompted us to revisit earlier steps rather than progressing linearly. For example, emerging findings required us to refine the programme theory to better integrate *Betti* into routine clinical workflows and to improve uptake and usability for both physicians and patients.

Prior work shows that the impact of digital health information and decision-support tools depends on consultation integration: e.g., in an RCT of a web portal for back pain patients rated physicians’ communication and consultation satisfaction worse in the intervention group using the web portal. Yet, only when physicians did not use the portal during the visit; when integrated into the encounter, no disadvantage was observed [40]. These results highlight that digital tools may undermine communication if they are merely provided to patients without being meaningfully incorporated into the consultation by physicians.

This finding was highly relevant for our development work: GPs in our proof-of-concept test expressed similar concerns regarding the challenge of integrating *Betti* into routine consultations. In response, we refined the intervention to strengthen the interface between the physician- and patient-facing components, enhance ease of use during live consultations, and create clearer opportunities for joint interaction with the material. Importantly, we also refined the programme theory accordingly, explicitly positioning consultation-integrated delivery as a core mechanism to improve communication, foster shared understanding and reassurance, and thereby support the intended impact.

### Implications for implementation

Our findings suggest an internalisation phenomenon, whereby GPs may learn *Betti’s* decision logic and consequently reduce or stop active tool use over time, whereas effects on imaging appropriateness may be sustained. A similar pattern was observed in our prior work on the decision aid “arriba Diabetes”: GPs valued the tool highly, but anticipated that they might use it less over time while still perceiving an enduring benefit through its educational function [55]. This pattern is echoed in other medical contexts, where earlier exposure to an intervention appears to train physicians, such that the tool use declines while practice change persists [56, 57].

These findings are also underpinned by the Normalisation Process Theory [58]. With respect to reflexive monitoring, the internalisation phenomenon suggests that, once physicians feel confident applying the logic without the tool, they may judge continued use as less necessary and may not perceive the intervention’s effects in day-to-day practice. At the same time, in terms of coherence, our data indicate that *Betti* is experienced as a credible and sensible approach—target user groups largely perceived it as a “good idea”—which may support endorsement and continued application of its recommendations even when observable tool use declines.

Therefore, a decline in *Betti* use in a physician’s subsequent consultations should not necessarily be interpreted as failure. If physicians internalise the decision logic and communication approach imaging appropriateness may still improve. In this scenario, success should be defined by sustained de-implementation outcomes (e.g., fewer inappropriate imaging requests, appropriate safety-netting, and preserved patient reassurance) rather than by use frequency alone. However, declining use may still be problematic if it weakens consultation-integrated delivery of the patient-facing components, as our proof-of-concept test indicated that patient materials were not accessed without physician-mediated handover. This should be investigated in a feasibility study or process evaluation of a randomised controlled trial evaluating *Betti*.

In line with Proctor’s implementation outcomes taxonomy our early-stage findings provide initial signals regarding acceptability, adoption, appropriateness, feasibility, and fidelity that are relevant for the further development and future implementation of *Betti* [29, 30]. In the proof-of-concept test and expert feedback, acceptability and perceived appropriateness were generally high, whereas feasibility constraints (time burden and workflow fit) and a marked adoption gap for patient-facing materials indicated that benefits are unlikely to be realised through passive provision alone. This pattern is consistent with wider evidence that digital decision support system and de-implementation interventions require careful embedding into routine work, and that multifaceted strategies are commonly needed to reduce low-value care [59–61].

### Strengths and limitations

This study has several strengths: First, *Betti* was developed systematically, drawing on the current MRC framework, an explicit logic model and the BCW as theoretical background. Second, the intervention is both multi-component and multi-target, combining GP training, decision support system and multimedia patient information. To our knowledge, *Betti* is also the first intervention explicitly designed to support appropriate imaging decisions across several common musculoskeletal pain presentations rather than focusing on a single region such as low back pain. Third, development was informed by a broad group of stakeholders—including GPs, specialists and patient representatives—and refined iteratively using data from a literature review, expert meetings, qualitative interviews and a proof-of-concept test. Finally, implementation and workflow fit were considered right from the outset.

This study also has limitations: First, *Betti* is yet at the stage of development and the proof-of-concept test involved a very small sample. In addition, the proof-of-concept sample was of limited diversity, as it included only four patients, all of whom were male, which may restrict the transferability of the findings to broader patient populations. Fidelity and adherence were explored through post-consultation qualitative interviews with GPs and patients. As intervention delivery was not directly observed, these findings reflect perceived rather than objectively verified implementation. Our narrative review did not include a formal quality appraisal. As a result, the review does not allow firm conclusions regarding the strength of the underlying evidence. The expert group included both clinicians and patient representatives. While this mixed composition was valuable in incorporating diverse perspectives, it may also have shaped the discussions despite the steps taken to mitigate potential power imbalances. The effectiveness of *Betti* in reducing unnecessary imaging and improving patient and economic outcomes has not yet been established and will need to be tested in future feasibility and effectiveness studies. Furthermore, the multicomponent nature of *Betti* and indications of additional consultation time and challenges to integrate the *Betti* intervention into the consultation suggest that implementation in routine care may be challenging.

### Implication for research and clinical practice

Future research should evaluate *Betti* using a staged approach (feasibility trial followed by an RCT with process evaluation) with simultaneous assessment of effectiveness and implementation outcomes. Outcomes should capture de-implementation of low-value imaging beyond tool use alone, including sustained practice change when decision support system use declines. An economic evaluation should be embedded to estimate implementation costs and potential savings from avoided inappropriate imaging. Beyond *Betti*, future research should investigate consultation communication as a key for de-implementation across low-value care. This could include developing and testing scalable, consultation-integrated communication supports that help physicians address expectations, uncertainty, and perceived medico-legal risk while maintaining patient reassurance and shared understanding.

Regarding implications for clinical practice, the three modules of *Betti* can be used in routine primary care to support appropriate imaging decisions for musculoskeletal pain. Furthermore, *Betti* may also be applicable in other settings where musculoskeletal imaging is discussed (e.g., orthopaedics). In addition, *Betti* may be useful as a teaching resource for GP trainees and senior medical students to develop skills in guideline-consistent imaging decisions and patient communication.

## Conclusions

We developed systematically and tested *Betti*, a multicomponent, consultation-integrated intervention to support appropriate imaging decisions for musculoskeletal pain in primary care. *Betti* addresses key determinants of low-value imaging by combining clinical decision support with structured communication and patient information materials. The proof-of-concept indicated that the intervention is feasible to use, but highlighted consultation-integrated delivery of patient materials as critical for achieving impact. *Betti* provides a transferable approach to de-implementing low-value imaging and offers a foundation for future feasibility and effectiveness evaluations, including economic assessment.

## Electronic Supplementary Material

Below is the link to the electronic supplementary material.

Supplementary Material 1

Supplementary Material 2

Supplementary Material 3

Supplementary Material 4

Supplementary Material 5

Supplementary Material 6

## Data Availability

All data generated or analysed during this study are included in this published article and its supplementary information files.

## Abbreviations

Betti: Better Imaging
BCW: Behaviour-Change-Wheel
CME: Continuing Medical Education
GP: General practitioner
MRC: Medical research council
RCT: Randomised Controlled Trial
TiDieR: Template for intervention description and replication
