# Supplementary material for: Development and proof-of-concept of a complex intervention to support appropriate imaging for musculoskeletal pain: the Betti programme
Source: Implement Sci Commun. 2026 May 5;7:88. doi: 10.1186/s43058-026-00949-4 (PMC13151194; doi:10.1186/s43058-026-00949-4)
Supplement: Supplementary file 1 — Supplementary Material 1 [file 43058_2026_949_MOESM1_ESM.docx]

**Supplement 1: Search strategy for narrative reviews**

**Narrative Review 1 on existing interventions on reducing inappropriate imaging in musculoskeletal pain**

Search Terms according to the PICO (Population, Intervention, Outcome) structure. Search terms of the columns were combined with AND.

Searches were performed in MEDLINE via PubMed

| **Population** | **Intervention** | **Outcome** |
| --- | --- | --- |
| “musculoskeletal pain” OR “back pain” OR “low back pain” OR “musculoskeletal disorders” OR “hip pain” OR “knee pain” OR “foot pain” OR “neck pain” OR “shoulder pain” OR “leg pain” OR “Osteoarthritis” OR “Tendinitis” OR “ankle pain” | “decision aid” OR “decision support system” OR “CDSS” OR “shared decision making” OR “SDM” OR “patient-centred care” OR “education” OR “digital health intervention” OR “Healthcare” OR “evidence-based medicine” OR “practice patterns” | “imaging reduction” OR “radiology referral” OR “communication improvement” OR “reduced imaging” OR “appropriate imaging” OR “inappropriate imaging” OR “unnecessary imaging” OR “communication” OR “satisfaction” OR “empowerment” OR “guideline adherence” OR “support evidence-based” OR “statistics & numerical data” OR “tomography” OR “magnetic resonance imaging” OR “x-ray computed” |

**Narrative Review 2 on guidelines on imaging for musculoskeletal pain**

Searches were performed in the following databases and additional platforms:

Trip database, AWMF database, NICE-guidelines, BMJ Best Practice, Royal College of General Practitioners, American College of Radiology

Search terms

Elbow: epicondylitis lateralis & medialis (lateral & medial epicondylitis), olecranon bursitis (aseptic & septic), thoracic outlet syndrome, radial tunnel syndrome/Syndrom, supinator syndrome/Syndrom, entzündlich rheumatische Erkrankungen (inflammatory arthritis), rheumatoide Arthritis (rheumatoid arthritis), Spondyloarthopathien (spondyloarthritis), axSpa, malignant disease elbow, bösartige Erkrankungen Ellenbogen

Lumbar spine: akute Rückenschmerzen (acute lower back pain), nicht spezifischer Rückenschmerz (non specific lower back pain), chronischer Rückenschmerzen (chronic lower back pain),  entzündlich rheumatische Erkrankungen (inflammatory arthritis), Spondyloarthopathien (spondyloarthritis), axSpa, malignant disease lower back/spine bösartige Erkrankungen Lendenwirbelsäule/Wirbelsäule, Trauma Lendenwirbelsäule (traumatic lower back pain), Iliosakraldysfunktion (sacroiliac joint dysfunction), Iliosakralgelenksschmerzen (sacroiliac joint pain)

Shoulder: Schulterschmerzen (general shoulder pain), Rotatorenmanschettenverletzung (rotator cuff tear/injury), acromioclaviculargelenk Schmerzen (acromioclavicular joint pain), adhäsive Kapselentzündung Schulter adhesive capsulitis shoulder), Schulter Bursitis (shoulder bursitis), glenohumeralgelenksschmerzen (glenohumaral joint pain), glenohumeralGelenk Arthrose (glenohumeral degenerative joint disease), thoracic outlet syndrome, polymyalgia rheumatica, entzündlich rheumatische Erkrankungen (inflammatory arthritis), rheumatoide Arthritis (rheumatoid arthritis), Spondyloarthopathien (spondyloarthritis), axSpa, malignant disease shoulder, bösartige Erkrankungen Schulter/Schultergelenk

Cervical spine: non-specific neck pain, Halswirbelsäulenarthrose, degenerative Veränderung Halswirbelsäule (cervical degenerative disc disease), cervical spondylosis, Bandscheibenveränderung Halswirbelsäule, Bandscheibenvorfall Halswirbelsäule, disc prolaps, disc degeneration, Schleudertraume (whiplash), cervical strain, cervical sprain, Facettengelenksarthrose, Facettengelenkstrauma, cervical facet joint syndrome, Totticollis, Radikulopathie Halswirbelsäule (cervical radiculopathy), cerival myelopathy, Fibromyalgie (fibromyalgia), chronisches Schmerzsyndrom (chronic pain syndrome), Spondylodiscitis, Polymyalgia rheumatica, entzündlich rheumatische Erkrankungen (inflammatory arthritis), Spondyloarthopathien (spondyloarthritis), axSpa,malignant disease cervical spine, bösartige Erkrankungen Halswirbelsäule

Hip: atraumatische Hüftkopfnekrose (atrumatic femoral head necrosis), bursitis trochanterica (trochanteric bursitis), inguinale Hernie (inguinale hernia), Hüftgelenksarthrose (hip joint osteoarthritis, degenerative joint disease hip), hip impingement (Cam, pincer), labral tear, meralgia paraesthetica, entzündlich rheumatische Erkrankungen (inflammatory arthritis), rheumatoide Arthritis (rheumatoid arthritis), Spondyloarthopathien (spondyloarthritis), axSpa, malignant disease hip, bösartige Erkrankungen Hüfte/Hüftgelenk

Hand: Handgelenksarthrose, Rizarthrose, Heberdens Arthose, osteoarthritis hand/wirst, DIPs/PIPs, Tendinose/Tendinopathie (tendinitis), de Quervains, Fraktur Os scaphoideum (scaphoid fracture), Dupuytrens-Kontraktur (Dupuytrens tendinopathy), schnellender Finger (trigger finger), Impingement nervus ulnaris/radialis (ulnar/radial nerve impingement), Karpaltunnelsyndrom (carpal tunnel syndrome), septische Arthritis (septic arthritis), thoracic outlet syndrome, entzündlich rheumatische Erkrankungen (inflammatory arthritis), rheumatoide Arthritis (rheumatoid arthritis), Spondyloarthopathien (spondyloarthritis), axSpa, malignant disease wrist/hand, bösartige Erkrankungen Hand/Handgelenk

Inclusion criteria: any clinical guideline or other professional resource (consensus or evidence based)

Relevant information on imaging were extracted in a word document
