# Supplementary material for: Development and proof-of-concept of a complex intervention to support appropriate imaging for musculoskeletal pain: the Betti programme
Source: Implement Sci Commun. 2026 May 5;7:88. doi: 10.1186/s43058-026-00949-4 (PMC13151194; doi:10.1186/s43058-026-00949-4)
Supplement: Supplementary file 2 — Supplementary Material 2 [file 43058_2026_949_MOESM2_ESM.docx]

**Supplement 2 Outline of topics and illustrative questions used in the practice test interview guide**

| **Interview guide for GPs** |
| --- |
| Overall impression  *What is your overall impression of Betti?*  *What changed through counselling with Betti?* |
| Functions of the Betti programme  *What did you like/dislike?*  *Which functions did you use, and why/why not?* |
| Implementation  *Can you imagine using Betti regularly?*  *What currently prevents you from using it?* |
| Closing  *In your view, when is Betti a success?*  *Is there anything else you would like to add?* |
| **Interview guide for patients** |
| Overall impression  *What was your impression of the consultation?*  *What is your overall impression of the materials?* |
| Message  *What is your opinion on imaging (X-ray, MRI) for musculoskeletal pain?*  *Did the consultation or the materials change your attitude?* |
| Functions of the Betti programme  *What did you like/dislike, and why?*  *What bothered you, and why?* |
| Implementation  *How can we best make these materials available to other patients* |
| Closing  *In your view, when is Betti a success?*  *Is there anything else you would like to add?* |
