# Supplementary material for: Development and proof-of-concept of a complex intervention to support appropriate imaging for musculoskeletal pain: the Betti programme
Source: Implement Sci Commun. 2026 May 5;7:88. doi: 10.1186/s43058-026-00949-4 (PMC13151194; doi:10.1186/s43058-026-00949-4)
Supplement: Supplementary file 3 — Supplementary Material 3 [file 43058_2026_949_MOESM3_ESM.docx]

Supplementary Material 3: Data Structure and Decision Logic

### Data structure as an operationalisation of clinical reasoning

The clinical decision support component of Betti is underpinned by an explicit, structured data model that operationalises guideline-based clinical reasoning. Clinical reasoning is not hard-coded as a fixed procedural algorithm or scoring system, but is represented as an explicit ontology of clinically meaningful considerations and relations, stored in relational tables. This declarative representation allows decision logic to be adapted and extended without restructuring the underlying program logic, while preserving a transparent, rule-based sequence of conditional considerations.

Each element of the decision logic corresponds to a distinct clinical consideration (e.g. assessment, differentiation, or conclusion) and is characterised by attributes that define its role within the overall reasoning process. These attributes specify, for example, whether a decision element represents a terminal point of the decision process, which type of user input is required, and whether additional explanatory information is available. In this way, the data structure governs both the logical progression of the decision support and the presentation of information to users, without producing automated recommendations or rankings.

By translating clinical reasoning into an explicit and inspectable structure, the data model supports transparency, traceability, and iterative refinement of the decision logic. At the same time, it preserves clinical discretion by structuring considerations rather than determining decisions.

### Managing decision tree complexity and transparency

During early development, the decision logic was conceptualised as a purely path-based, state-free decision tree, in which all relevant contextual information was encoded exclusively in the decision path, such that each user response led to a subsequent state. This approach was initially chosen to ensure conceptual clarity and traceability of the decision process.

However, during implementation it became apparent that a strictly path-based representation led to rapid growth in the size and complexity of the decision tree. As additional clinical distinctions and contextual factors were incorporated, the resulting structure became increasingly difficult to inspect, discuss, and maintain. This posed a challenge given that the decision logic was intended to remain transparent and cognitively accessible, not only functionally correct.

To address this, additional state variables were introduced and passed alongside the decision path. This allowed relevant contextual information to be retained without further branching of the tree. As a result, structural complexity was substantially reduced while preserving explicit rule-based logic. Importantly, this design adjustment improved readability and maintainability without introducing opaque or automated decision-making mechanisms.

This experience highlights the importance of aligning technical representations of decision logic with goals of transparency, cognitive accessibility, and implementation feasibility. Design decisions at the level of data structures can therefore have substantial implications for how decision support systems are understood, scrutinised, and used in practice.

The use of a relational data model with distinct entity types further supports this flexibility, allowing individual components of the decision logic to be modified, recombined, or extended without restructuring the entire system.

### Automated generation of multiple representations of the decision logic

The decision logic underlying Betti is stored in a relational, machine-readable format that serves as a *single source of truth* for the intervention. This design allows the same underlying data structure to be used to generate different representations of the decision logic automatically.

From this shared data source, the system renders the interactive decision support used during consultations, generates graphical visualisations of the decision logic as decision trees, and produces a table-based representation of the logic that can be exported as a printable PDF for offline use. The tabular format follows an explicit if–then structure with references to subsequent steps, allowing the decision logic to be inspected and used independently of the digital interface.

Given the scope of the decision logic and its iterative refinement during development, maintaining these representations manually would not be feasible. Automatic generation ensures consistency across representations, supports transparency, and allows updates to propagate reliably across formats without additional manual effort.
