# Supplementary material for: Development and proof-of-concept of a complex intervention to support appropriate imaging for musculoskeletal pain: the Betti programme
Source: Implement Sci Commun. 2026 May 5;7:88. doi: 10.1186/s43058-026-00949-4 (PMC13151194; doi:10.1186/s43058-026-00949-4)
Supplement: Supplementary file 4 — Supplementary Material 4 [file 43058_2026_949_MOESM4_ESM.docx]

|  | **Study** | **Focus** | **Design** | **Procedures** | **Betti-relevant findings** | **Betti integration** |
| --- | --- | --- | --- | --- | --- | --- |
| patients | | | | | | |
| 1 | Djurtoft et al 2025^1^ | Low back pain  patients | multi-method: systematic literature search, input from practice consultants, development of a program theory, interview study, with people experiencing low back pain, consensus meeting | Creation of a leaflet (Choosing Wisely) supporting reduction of imaging usage in low back pain | It was recommended that the leaflet targeted people who were at risk of poor prognosis, such as having negative thoughts about low back pain and its management. | In the training material, physicians are advised to specifically engage patients at elevated risk of a poor prognosis. |
|  |  |  |  |  | Furthermore, it was highlighted that the leaflet should be designed to be distributed by healthcare professionals with additional explanations, made available online, and possibly supplemented with QR codes linking to other materials, such as informational videos. | The patient information material consists of a brochure and additional online multimedia material |
| 2 | Sharma et al. 2021^2^ | Back pain  patients | controlled experimental study | waiting room communication strategy, designed to raise awareness of potential harms of unnecessary imaging, on lumbar imaging  rates in the emergency department (ED)  result: may slightly reduce the proportion of patients with low back pain who receive lumbar imaging | Use of four languages | plan to include other languages in the Betti programme |
| 3 | Simula et al. 2021^3^ | cluster-randomized controlled trial | low back pain  patients  primary care | patient-education booklet  providing practitioners with the new patient-education booklet and a 30-min training session on its use  Using the patient education booklet appeared to substantially reduce the proportion of LBP patients who underwent an imaging examination at three months, but the result was not statistically significant | Presentation of the patient-education booklet | Discussion of integration of parts of the booklet into the Betti patient material, found as not appropriate for different types of musculoskeletal pain  Discussion on the use of the term “serious” as patients may misinterpret the term serious |
| 4 | Egerton et al. 2021^4^ | Knee pain  patients | mixed methods design, including a survey and semi-structured interviews | Participants reviewed an educational video on kee osteoarthritis | The educational video about knee osteoarthritis with a focus on empowerment is well received. | Ideas to improve the Betti video for patients |
| 5 | Suman et al 2017^5^ | Low back pain  patients | process evaluation using a mixed methods approach | Guided website with information, videos, exercises, newsletters; social media; partial translations; access via HCPs; assessment of reach, dose delivered/received, satisfaction, barriers | Actual use was low (many patients visited once or not at all); satisfaction increased with more use; patients wanted earlier access (at first low back pain episode), more attractive layout, and clinician endorsement; clinicians rarely discussed the campaign, which limited trust and uptake | Betti should be introduced and endorsed by GPs during the consultation; access should be simple and early; design needs to be concise and attractive; offer printable/downloadable patient material; consider translations for relevant groups to broaden reach. |
| 6 | Sharma et al. 2021^6^ | Low back pain  patients | Three-arm online randomised superiority trial | Participants viewed one leaflet: Standard Care (guideline info), Neutral (balanced benefits/harms Fact Box), or Nudge (behavioural-economics cues: framing, loss aversion, anchoring, chunking, status-quo, alternatives, cue-to-action). | intention to request imaging was lower in the *Nudge* Leaflet group | The patient information material is clear and easily readable. Discussion if nudging is ethical correct, therefore not directly included into Betti. However, we did include parts e.g. cue to action. |
| Healthcare professionals | | | | | | |
| 7 | Fenton et al. 2024^7^ | Lumbar spinal imaging  GPs | RCT | communication intervention  GPs of the intervention group received 3 simulated office visits with a simulation patient potraying a patient with acute uncomplicated back pain: (1) set the stage for deferred imaging by building trust, (2) convey empathy, and (3) communicate optimism while advocating watchful waiting without imaging.  Feedback guided by a 3-step model  No difference of primary outcome: patients with acute low back pain who saw intervention and control clinicians during follow-up had similar rates of lumbar imaging | Presentation of communication strategy in three steps | Ideas for communications strategy for the training material for clinicians. |
|  |  |  |  |  | Intervention only targeting GPs was not effective | Betti targets clinicians and GPs. |
| 8 | Baker et al. 1987^8^ | Acute back pain  Clinicians working in an emergency department | Prospective controlled before-and-after study | emergency  room house officers were encouraged to complete a special form that listed only three  acceptable indications for lumbosacral spine radiographs: history of trauma, evidence  of focal neurologic abnormality, and “other” (other combined with free text)  seems to be effective: In the control year 1443 examinations  were performed, and in the experimental year only 759 were done (a decrease of 47%). | NA | NA |
| 9 | Buller-Close et al 2003^9^ | Back pain  clinicians | Prospective staggered off–on–off controlled time-series experiment | Tested an electronic expert charting system (EDECS) in the emergency department. The low back pain module provided guideline-based prompts, documentation, and discharge instructions to clinicians during patient encounters  Improved documentation and guideline adherence for back pain, but effects were less pronounced compared to rarer conditions | Decision support with guideline-based recommendations and generation of structured documentation. | The decision support system of Betti provides guideline-based recommendations, documentation, and follow-up instructions |
| 10 | Day et al. 1995^10^ | Back pain  Physicians working in an emergency department | Prospective controlled time-series (before–after) study | The physician, aided by a list of "essential" items required by the computer, interviews and examines the patient. The intervention then analyzes the complete database and suggests appropriate tests and  treatments.  Little effect on appropriateness of imaging or costs | Presentation of a checklist-guided diagnostic way | Discussion in the second expert meeting to provide a diagnostic algorithm in form of a checklist. However, idea was disregarded as it not proven feasible for all types of musculoskeletal pain. Instead we provide examination tips incorporated into the decision support system. |
| 11 | Peters et al. 2022^11^ | Low back pain  Clinicians working in an emergency department | uncontrolled before-and-after design | Development of an **evidence-based triage protocol** for low back pain and radicular pain.  Implementation strategies included: staff education sessions, patient brochures, symposium and letters for GPs, audit and feedback, and later compulsory e-learning and posters to address sustainability challenges  Result:  The rate of imaging of the lumbar spine decreased from over 25% of patients to 15.0%–16.4% for CT scans and 19.0%–21.8% for X-rays after implementation, but started to fluctuate again after 3 years.  Implementation of a new protocol in a tertiary hospital ED with high turn over of rotating trainees is a challenge and requires ongoing efforts to ensure sustainability. | NA | NA |
| 12 | Min et al 2017^12^ | Back pain  Clinicians working in an emergency department  patients | Prospective single-center pre–post intervention study | Embedded a checklist of accepted red flags for low back pain into the electronic order entry form for lumbar imaging in a large urban emergency department. Physicians had to indicate red flags or provide justification before ordering imaging; patients received educational material.  Imaging orders for low back pain decreased significantly (median 22% → 17%) without an increase in missed serious diagnoses or ED revisits | point-of-care decision support integrated into clinical workflow can safely reduce unnecessary imaging | Not possible to integrate into the Betti decision support system |
| 13 | Tun et al 2023^13^ | Low back pain  Physicians  Primary care | pilot cluster randomized controlled trial with qualitative interviews | electronic decision support system for LBP (DeSSBack) was developed based on an evidence-based risk stratification tool to improve the management of patients with LBP | NA | NA |
| 14 | Burgon et al 2021^14^ | Primary care physicians (various conditions including musculoskeletal pain) | Randomised controlled trial | Physicians completed online case simulations covering typical primary care scenarios (e.g. diabetes, OA, asthma, musculoskeletal pain pain). Each case provided immediate evidence-based feedback  Results: Evidence-based care decisions improved significantly, CME availability did not affect performance | Presentation of an online case simulation for musculoskeletal pain | Not incorporated into Betti as the Betti programme is not provided in form of a course covering multiple weeks |
| 15 | Fourré et al. 2022^15^ | Different health care professionals  Back pain | Feasibility study | interactive e-learning module on the management on the management of back pain  e-learning intervention included written parts, voice-overs and clinical encounter videos | NA | NA |
| 16 | Blackmore et al. 2011^16^ | Low back pain  Physicians working in a multidisciplinary health care net-  work | Retrospective cohort before–after study | Staged implementation of locally agreed evidence-based imaging rules mandatory indication check at order entry; denial of orders without approved indication; institutional education and periodic audits; offer of alternative same-/next-day specialist or physiotherapy consultations | Intervention led to a decrease of imaging. success depended on (i) being embedded in the actual ordering workflow, (ii) using locally agreed guideline criteria, and (iii) offering clinical alternatives so that “no imaging” did not block care | Integration of Betti into the practice software might be beneficial |
| 17 | Freeborn et al. 1997^17^ | Back pain  GPs | Controlled intervention (two medical areas: guideline ± feedback vs. control), pre–post using routine imaging data | Mailing of a comprehensive low back pain process-of-care guideline (incl. imaging algorithm), short presentations at department meetings, then three bimonthly feedback reports on each physician’s imaging rates with anonymised peer ranking; comparison with control area using automated radiology data**.** | Guideline plus feedback alone did not reduce imaging rates nor physician-to-physician variability; physicians said the material was useful, but contextual factors (time pressure, patient expectations, gatekeeper role) overruled the guideline; unfocused/too broad guidance may dilute impact on the single behaviour “no imaging” | Betti should deliver focused, consultation-near imaging guidance rather than broad documents; address patient expectations and GP time constraints explicitly; consider embedding Betti in routine workflow instead of relying on mailed guidelines and periodic reports alone. |
| 18 | Fenton et al. 2016^18^ | Low back pain  GPs | RCT | Two standardized patient instructor visits teaching a 6-step communication to elicit expectations, reassure, explain non-testing, and offer alternatives; control received SPs without feedback. | Brief, stand-alone communication training was well received but did not reduce actual ordering of low-value imaging. | Betti should not rely on one-off GP training alone; the “no imaging” message must be embedded in the real consultation and supported by concrete patient material. |
| 19 | Ip et al. 2014^19^ | Low back pain  GPs | Pre–post study | Implementation of a decision support system, mandatory peer-to-peer consultation when test utility was uncertain and quarterly practice pattern variation reports. | Intervention reduced imaging rate for low back pain. | Integration of Betti into the practice software might be beneficial. |
| 20 | Wang et al. 2018^20^ | Back pain  clinicians | Pre–post observational study over 2×10 months (before/after educational intervention) | Baseline extraction of all lumbar MRI orders, followed by two 1 h on-site education for clinicians on Choosing Wisely criteria with case vignettes, repeated extraction of MRI orders. | The education reduced MRI scans and increased appropriateness criteria. | NA |
| 21 | Suman et al 2018 ^21^ | Low back pain  GPs | before-after study | The implementation strategy consisted of amulticomponent, multidisciplinary continuing medical education (CME) training with focus on interdisciplinary communication and collaboration, patient-physician communication. Additional materials including online and offline educational materials, social media platforms. Supported by a patient-based eHealth strategy | Baseline imaging and referral rates were already low, the strategy did not lower imaging or total referrals beyond usual care | Discussion to target practices with a  higher baseline imaging rate to be able to see effects. |
| 22 | Osteras et al. 2021^22^ | hip and knee osteoarthritis  physiotherapists and GPs | Quasi-experimental interrupted time-series | Two workshops (PT and multidisciplinary GP/PT) plus a PT discharge-report template were part of the intervention.  The intervention intended to facilitate communication between health professionals in primary healthcare and with healthcare professionals in specialist healthcare to ensure that people with hip and knee OA would experience timely, well integrated, and high-quality OA care | To standardize the content and potentially reduce the time needed for PTs to write discharge reports, a template for OA discharge reports was developed by the project group and distributed to the PTs. | The decision support system of Betti provides a template for imaging referrals and a copy-pase text for a normal examination for the documentation. |
| 23 | Peiris et al 2014^23^ | Back pain  GPs | Development and mixed-methods evaluation | Development of an online tool structured around exclusion of serious pathology, assessment and management options; national roll-out via professional channels; collection of web analytics; qualitative interviews with GPs on usability and impact. | Tool in stepwise structure was generally acceptable to GPs; perceived usefulness varied by clinical context but was higher when the tool helped justify not proceeding to more complex diagnostics; and external dissemination (media release during Australian National Pain Week) clearly increased uptake. The patient information sheet was a particularly useful component, as it synthesized the key messages that would otherwise take some time to explain. | Betti also follows a step-wise procedure. We discussed to provide more patient specific information materials. We discussed to tie implementation to external events (e.g. national/back pain days). |
| Healthcare professionals and patients | | | | | | |
| 24 | Coombs et al. 2021^24^ | Low back pain  Clinicians working in an emergency department  patients | RCT | multifaceted intervention to implement guideline recommendations for low back pain care, with five main components:  1. education seminars on skills for assessing, managing, educating and referring patients with acute low back pain  2. Educational materials consisting of a hard copy of the model of care, a website and decision support tools for appropriate use of lumbar imaging and analgesic medicines and posters and patient information material on benefits and harms of lumbar imaging  3. Provision of non-opioid pain management strategies  4. Fast-track referral to outpatient services  5. Audit and feedback  Result: no clear evidence that the intervention reduced lumbar imaging | NA | NA |
| 25 | Haig et al. 2019^25^ | Back pain  Physicians and patients in an emergency department | Prospective interventional trial | Complex Consulting Process including coordinated  process of Emergency physician and patient education, standardized intake and order protocols, and rapid access to Physiatry and physical  therapy  patient  pamphlet was developed  Imaging orders did not change significantly | NA | NA |
| 26 | Sapadin et al. 2022^26^ | Back pain  Clinicians and patients  Emergency department | Prospective quality improvement (QI) interventional study (uncontrolled before–after) | multi-component intervention led by a clinician champion including staff education, patient education, electronic medical record modification, audit and peer-feedback, and clinical decision support tools  result: seems to be an effective way to reduce the overutilization of thoracic and lumbar radiographs | multi-component intervention seemed to be effective | We developed Betti in form of different modules. |
| 27 | Larijani et al. 2021^27^ | Lower back pain  Patients and clinicians | Qualitative development study | A lower back pain prescription pad was developed  participants reported a lack of interactive and informative communication was a significant barrier to receiving appropriate care.  Participants also suggested it was important that benefits and risks of imaging were explained on the pad.  Three key themes derived from the data were also used to guide development of the intervention: (a) the role of imaging in LBP diagnosis; (b) the impact of the patient-physician relationship on LBP diagnosis and treatment; and (c) the lack of patient awareness of Choosing Wisely Canada and their recommendations. | Most participants asserted their belief that having imaging was important for treatment of LBP, and they indicated that imaging freed them from the stress of not knowing what was wrong. | Patient information material of Betti has a focus that imaging should only be performed when it has a consequence. |
|  |  |  |  |  | In some cases, participants felt that imaging was ordered only because their doctor was rushed and only had time for a very short consultation.  Four patients’ experiences showed a lack of interactive and informative communication was a significant barrier to receiving appropriate care for LBP  Six out of nine patients mentioned a lack of clarity about “what happens next”. | Provision of communication strategies in the physician training material.  The importance of a clear plan and follow-up is emphasised in the physician training materials and the decision support system. |
| 28 | Traeger et al. 2020^28^ | Low back pain  Patients and clinicians | Qualitative study | GPs and patients reviewed three communication tools | GPs and patients agreed that a leaflet about overdiagnosis could support a delayed prescribing approach to imaging for low back pain. | Ideas for the Betti patient information material. Discussion on integration of a signed agreement, but disregarded as evidence of this study was mixed. |
| 29 | Morgan et al. 2019^29^ | Low back pain  GPs and patients | Retrospective population-based time-series evaluation with Bayesian structural time-series | The program delivered referral pattern feedback, a decision support tool and patient information. It was associated with a statistically significant 10.85% relative reduction in the volume of CT scans of the lumbosacral region | A complex intervention targeting GPs and patients with multiple compements seems to be effective. | The Bett programme targest GPs and patients and consists of different modules. |
| 30 | Voigt-Radloff et al 2019^30^ and other tala-med publications^31^ | Back pain  Physicians and patients | prospective multi-centre, cluster-randomized parallel group trial | GPs in the intervention group used and recommended tala-med during the consultation; patients then had access to the portal. | Tala-med improves patients’ informedness and acceptance of recommended management when they are actively used during the consultation, it requires good GP introduction to avoid poorer communication ratings. | The layout of the tala-med homepage informed the structure of Betti; at the same time, the tala-med studies shows that the tool only reaches patients effectively when GPs actively and properly introduce it during the consultation. |
| 31 | Zafar et al. 2019^32^ | Back pain  physicians | Pragmatic, practice-randomised 3-period study | Baseline: CDS classified MRI orders silently. Period 1: group received either periodic, provider-level report cards (with peer comparison) or real-time CDS alerts at order entry. Period 2: all practices received both report cards and real-time alerts. Orders within day 0 and days 1–30 after LBP visit were analysed with multilevel regression | Provider-level, periodic feedback with peer comparison achieved a marked reduction in potentially inappropriate lumbar MRI, whereas real-time alerts alone did not change ordering. | NA |
| 32 | Jenkins et al 2018 | Back pain  GPs and patients | Theory-driven intervention development using the Behaviour Change Wheel interviews and expert inputs | The Behaviour Change Wheel was used to identify the behaviours requiring change, and guide initial development of an implementation intervention. Preliminary testing of the intervention was performed with: 1) content review by experts in the field; and 2) qualitative analysis of semi-structured interviews with 10 GPs and 10 healthcare consumers, to determine barriers and facilitators to successful implementation of the intervention in clinical practice. | Overuse of imaging was linked to combined patient expectations and GP barriers. A brief, consultation-time resource that explains why imaging is not needed and that provides an individualised management plan was acceptable to both GPs and patients. | Betti is likewise underpinned by the Behaviour Change Wheel, offers a structured clinical resource for GPs, emphasises communication to reassure patients about guideline-concordant non-imaging management, and enables GPs to provide patient information/handouts as an alternative to ordering imaging. |

References

1. Djurtoft C, O'Hagan E, Laursen MD*, et al.* Co-creating a Choosing Wisely leaflet supporting the reduction of imaging usage in low back pain management - A multi-method study. *Patient Educ Couns* 2025; **135:** 108730.

2. Sharma S, Traeger AC, Tcharkhedian E*, et al.* Effect of a waiting room communication strategy on imaging rates and awareness of public health messages for low back pain. *Int J Qual Health Care* 2021; **33(4)**.

3. Simula AS, Jenkins HJ, Hancock MJ*, et al.* Patient education booklet to support evidence-based low back pain care in primary care - a cluster randomized controlled trial. *BMC Fam Pract* 2021; **22(1):** 178.

4. Egerton T, McLachlan L, Graham B*, et al.* How do people with knee pain from osteoarthritis respond to a brief video delivering empowering education about the condition and its management? *Patient Educ Couns* 2021; **104(8):** 2018–27.

5. Suman A, Schaafsma FG, Bamarni J*, et al.* A multimedia campaign to improve back beliefs in patients with non-specific low back pain: a process evaluation. *BMC Musculoskelet Disord* 2017; **18(1):** 200.

6. Sharma S, Traeger AC, O'Keeffe M*, et al.* Effect of information format on intentions and beliefs regarding diagnostic imaging for non-specific low back pain: A randomised controlled trial in members of the public. *Patient Educ Couns* 2021; **104(3):** 595–602.

7. Fenton JJ, Cipri C, Gosdin M*, et al.* Standardized Patient Communication and Low-Value Spinal Imaging: A Randomized Clinical Trial. *JAMA Netw Open* 2024; **7(11):** e2441826.

8. Baker SR, Rabin A, Lantos G*, et al.* The Effect of Restricting the Indications for Lumbosacral Spine Radiography in Patients with Acute Back Symptoms; **1987**.

9. Buller-Close K, Schriger DL, Baraff LJ. Heterogeneous effect of an Emergency Department Expert Charting System. *Ann Emerg Med* 2003; **41(5):** 644–52.

10. Day F, Hoang LP, Ouk S*, et al.* The impact of a guideline-driven computer charting system on the emergency care of patients with acute low back pain. *Proc Annu Symp Comput Appl Med Care* 1995: 576–80.

11. Peters S, Jacobs K, van Wambeke P*, et al.* Applying a knowledge translation framework for triaging low back pain and radicular pain at an emergency department: an iterative process within an uncontrolled before-and-after design. *BMJ Open Qual* 2022; **11(4)**.

12. Min A, Chan VWY, Aristizabal R*, et al.* Clinical Decision Support Decreases Volume of Imaging for Low Back Pain in an Urban Emergency Department. *J Am Coll Radiol* 2017; **14(7):** 889–99.

13. Tun Firzara AM, Teo CH, Teh SY*, et al.* Evaluation of an electronic clinical decision support system (DeSSBack) to improve low back pain management: a pilot cluster randomized controlled trial. *Fam Pract* 2023; **40(5-6):** 742–52.

14. Burgon T, Casebeer L, Aasen H*, et al.* Measuring and Improving Evidence-Based Patient Care Using a Web-Based Gamified Approach in Primary Care (QualityIQ): Randomized Controlled Trial. *J Med Internet Res* 2021; **23(12):** e31042.

15. Fourré A, Fierens A, Michielsen J*, et al.* An interactive e-learning module to promote bio-psycho-social management of low back pain in healthcare professionals: a pilot study. *J Man Manip Ther* 2022; **30(2):** 105–15.

16. Blackmore CC, Mecklenburg RS, Kaplan GS. Effectiveness of clinical decision support in controlling inappropriate imaging. *J Am Coll Radiol* 2011; **8(1):** 19–25.

17. Freeborn DK, Shye D, Mullooly JP*, et al.* Primary care physicians' use of lumbar spine imaging tests: effects of guidelines and practice pattern feedback. *J Gen Intern Med* 1997; **12(10):** 619–25.

18. Fenton JJ, Kravitz RL, Jerant A*, et al.* Promoting Patient-Centered Counseling to Reduce Use of Low-Value Diagnostic Tests: A Randomized Clinical Trial. *JAMA Intern Med* 2016; **176(2):** 191–7.

19. Ip IK, Gershanik EF, Schneider LI*, et al.* Impact of IT-enabled intervention on MRI use for back pain. *Am J Med* 2014; **127(6):** 512-8.e1.

20. Wang KY, Yen CJ, Chen M*, et al.* Reducing Inappropriate Lumbar Spine MRI for Low Back Pain: Radiology Support, Communication and Alignment Network. *J Am Coll Radiol* 2018; **15(1 Pt A):** 116–22.

21. Suman A, Schaafsma FG, van de Ven PM*, et al.* Effectiveness of a multifaceted implementation strategy compared to usual care on low back pain guideline adherence among general practitioners. *BMC Health Serv Res* 2018; **18(1):** 358.

22. Østerås N, Blaker IB, Hjortland T*, et al.* Improving osteoarthritis management in primary healthcare: results from a quasi-experimental study. *BMC Musculoskelet Disord* 2021; **22(1):** 79.

23. Peiris D, Williams C, Holbrook R*, et al.* A web-based clinical decision support tool for primary health care management of back pain: development and mixed methods evaluation. *JMIR Res Protoc* 2014; **3(2):** e17.

24. Coombs DM, Machado GC, Richards B*, et al.* Effectiveness of a multifaceted intervention to improve emergency department care of low back pain: a stepped-wedge, cluster-randomised trial. *BMJ Qual Saf* 2021; **30(10):** 825–35.

25. Haig AJ, Uren B, Loar S*, et al.* The Impact of a Complex Consulting Process with Physiatry on Emergency Department Management of Back Pain. *The Journal of the International Society of Physical and Rehabilitation Medicine* 2019; **2(2):** 77–87.

26. Sapadin J, Campbell L, Bajaj K*, et al.* Reducing thoracic and lumbar radiographs in an urban emergency department through a clinical champion led quality improvement intervention. *BMC Emerg Med* 2022; **22(1):** 69.

27. Madani Larijani M, Dumba C, Thiessen H*, et al.* Development of a Patient-Oriented Intervention to Support Patient-Provider Conversations about Unnecessary Lower Back Pain Imaging. *Int J Environ Res Public Health* 2021; **18(5)**.

28. Traeger AC, Checketts J, Tcharkhedian E*, et al.* Patient and general practitioner views of tools to delay diagnostic imaging for low back pain: a qualitative study. *BMJ Open* 2020; **10(11):** e039936.

29. Morgan T, Wu J, Ovchinikova L*, et al.* A national intervention to reduce imaging for low back pain by general practitioners: a retrospective economic program evaluation using Medicare Benefits Schedule data. *BMC Health Serv Res* 2019; **19(1):** 983.

30. Voigt-Radloff S, Schöpf AC, Boeker M*, et al.* Well informed physician-patient communication in consultations on back pain - study protocol of the cluster randomized GAP trial. *BMC Fam Pract* 2019; **20(1):** 33.

31. Schlett C, van der Keylen P, Schöpf-Lazzarino AC*, et al.* The Effectiveness of a Physician-Led Web Portal on Back Pain: A Cluster Randomized Controlled Trial. *Dtsch Arztebl Int* 2025; **122(8):** 203–9.

32. Zafar HM, Ip IK, Mills AM*, et al.* Effect of Clinical Decision Support-Generated Report Cards Versus Real-Time Alerts on Primary Care Provider Guideline Adherence for Low Back Pain Outpatient Lumbar Spine MRI Orders. *AJR Am J Roentgenol* 2019; **212(2):** 386–94.
