# Supplementary material for: Development and proof-of-concept of a complex intervention to support appropriate imaging for musculoskeletal pain: the Betti programme
Source: Implement Sci Commun. 2026 May 5;7:88. doi: 10.1186/s43058-026-00949-4 (PMC13151194; doi:10.1186/s43058-026-00949-4)
Supplement: Supplementary file 5 — Supplementary Material 5 [file 43058_2026_949_MOESM5_ESM.docx]

**Supplement 4 Impression of Betti**

[www.entscheidung-bildgebung.de](http://www.entscheidung-bildgebung.de)

**
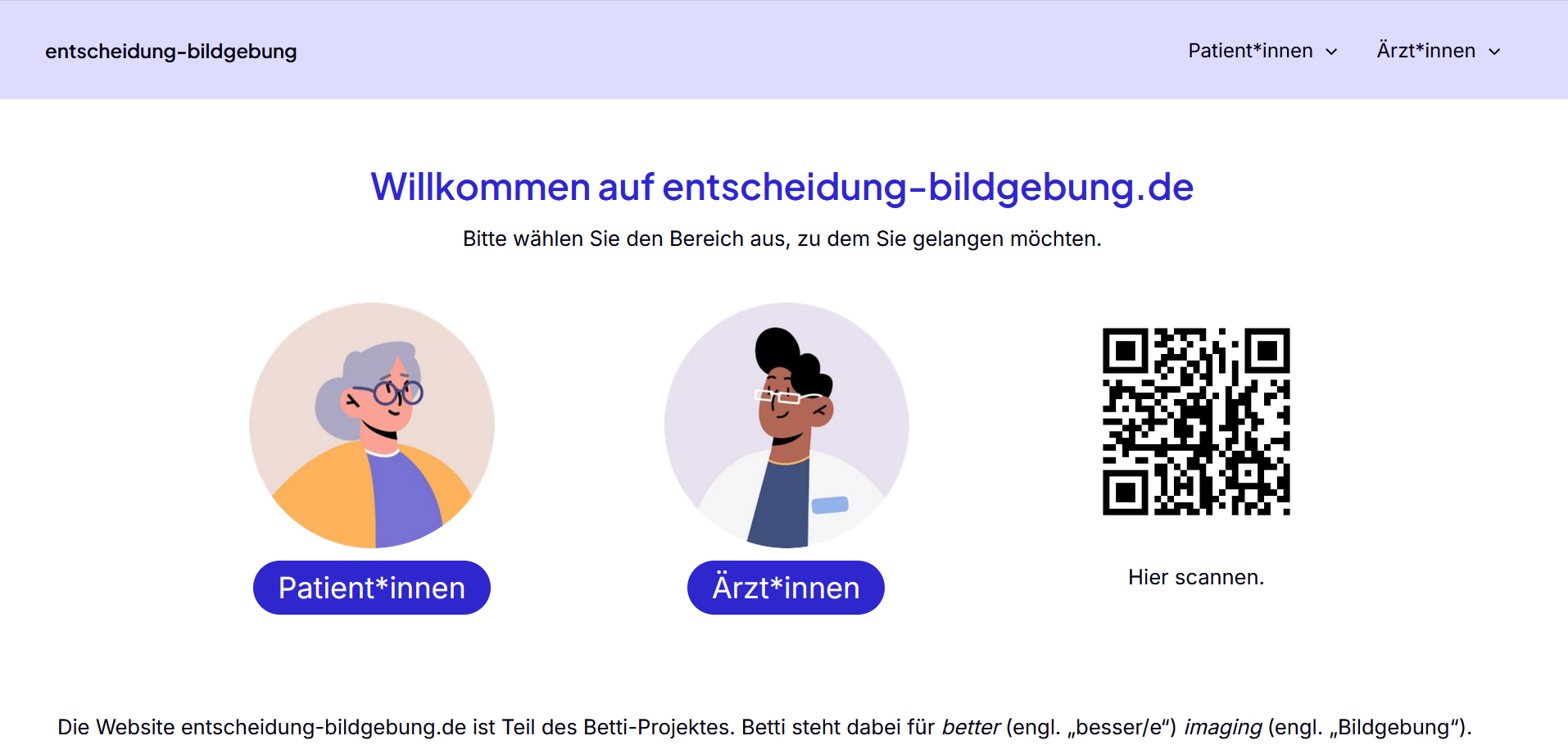
**

**
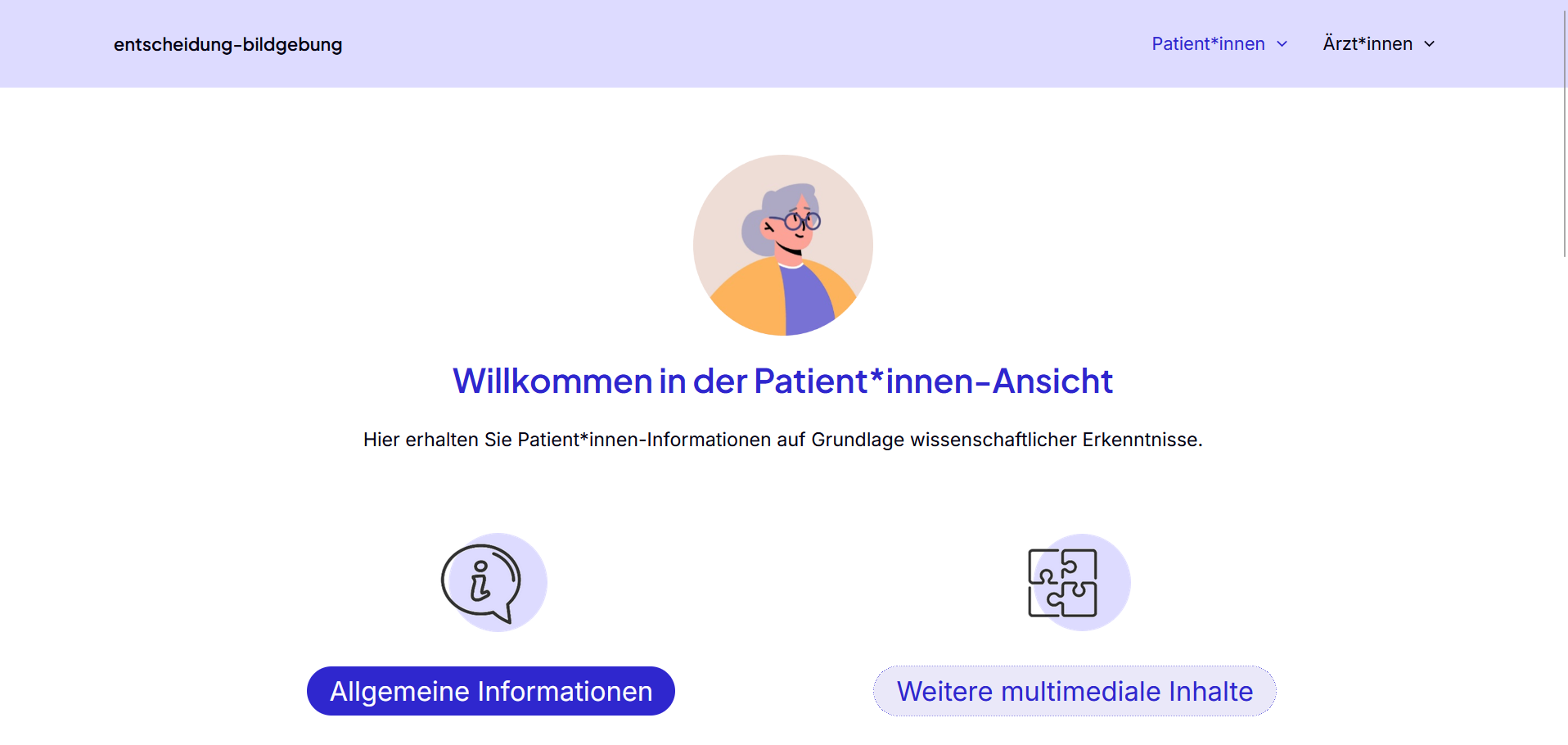
**

**
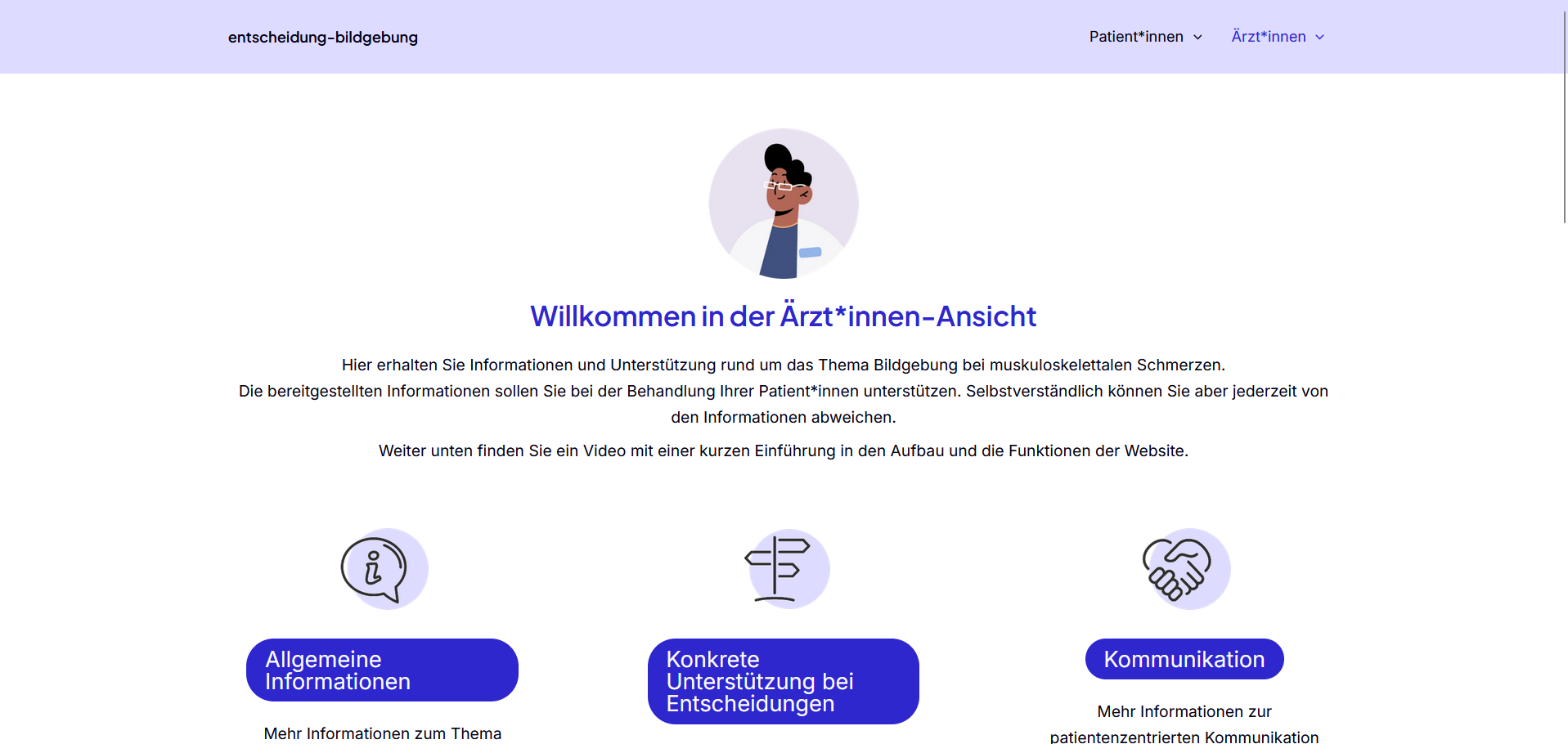
**

**
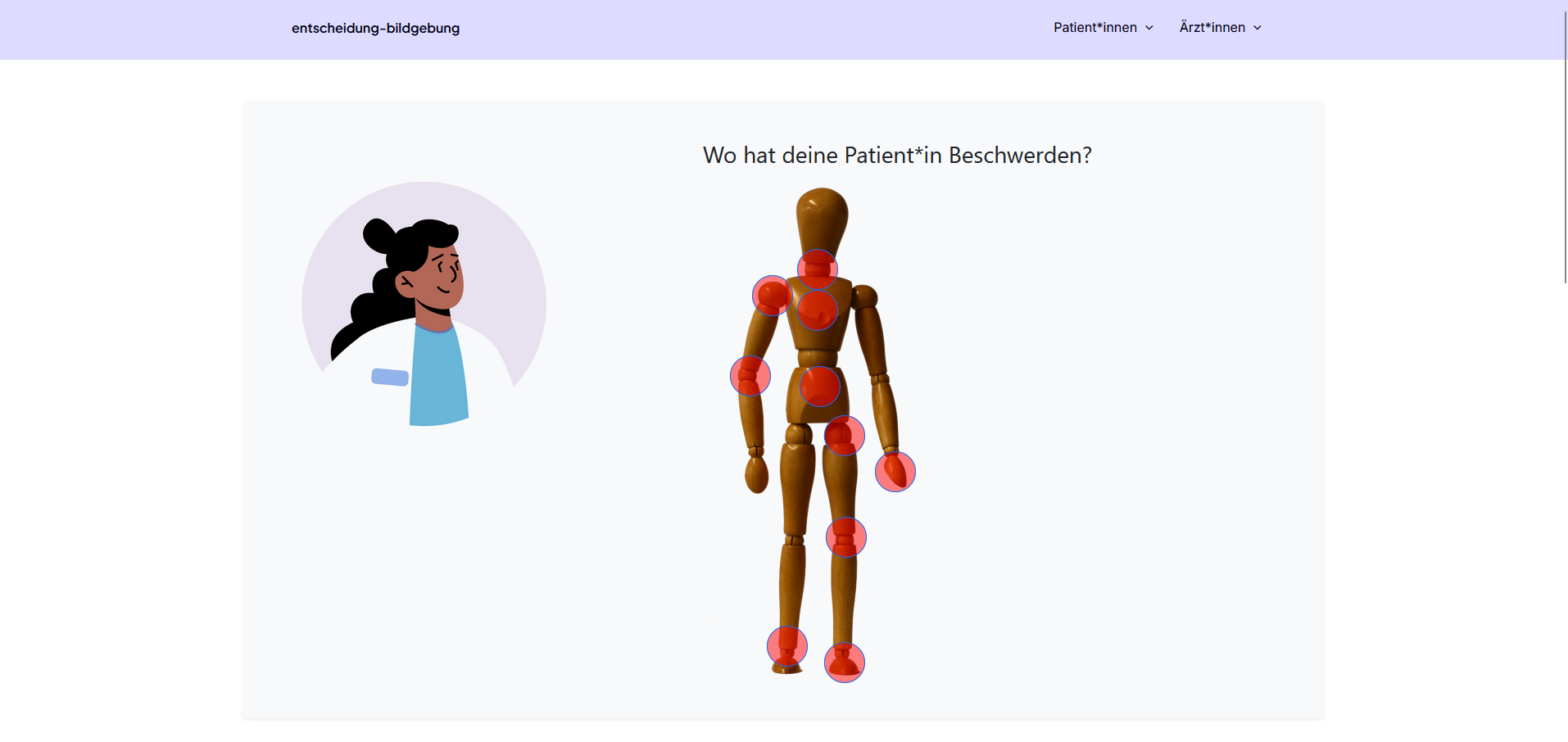
**
