# Supplementary material for: Development and proof-of-concept of a complex intervention to support appropriate imaging for musculoskeletal pain: the Betti programme
Source: Implement Sci Commun. 2026 May 5;7:88. doi: 10.1186/s43058-026-00949-4 (PMC13151194; doi:10.1186/s43058-026-00949-4)
Supplement: Supplementary file 6 — Supplementary Material 6 [file 43058_2026_949_MOESM6_ESM.docx]

**Supplement 5 Plan for future implementation**

Table 1 Supplement 5 Early-stage implementation outcomes for Betti and resulting targets for refinement

| **Implementation outcome** | **Considerations to support later implementation** | **Implications/ targets for refinement** |
| --- | --- | --- |
| Acceptability | High among GPs and patients; Betti perceived as relevant, well structured, easy to understand, and supportive of consultation communication. Experts rated the CDSS as helpful/accurate/practicable and patient-facing components positively | Maintain current format and messaging |
| Adoption | Marked difference between clinician and patient components: no patients viewed patient materials before interview (likely because of missing GP–patient handover). One GP used Betti outside the study context. | Strengthen clinician-mediated handover (incorporated as output in the logic model and refinement) to increase access to patient materials |
| Appropriateness | Generally supported: perceived as practice-oriented and aligned with routine care. Requests for more condition-specific visuals/explanations. | Add as much condition-specific information as feasible for patients, stating why imaging is recommended |
| Feasibility | \| Main challenges: time burden and limited workflow integration; missing interface to patient materials. Experts recommended streamlined processes and modular formats. \| \| --- \| | Reduce time costs through streamlined navigation and modular content |
| Fidelity | Dependent on consultation-integrated delivery and GP–patient linkage to patient materials; linkage not achieved in the practice test. | Emphasise consultation-integrated tool use and a reliable GP–patient linkage to the patient material as output in the logic model  Build interface for frictionless transfer to patient material |
| Implementation costs | Primarily additional clinician time. Potential system-level savings if Betti reduces cost-intensive low-value imaging. | Planned: Include economic evaluation (time costs vs avoided imaging and downstream costs) in future studies |
| Penetration | Planned via multi-channel dissemination: CME-accredited training, external courses/GP platforms, mobile-friendly website; patient reach via media/social media and practice materials (posters/brochures, plain-language). (See Table 5) | Planned: Monitor use in future studies |
| Sustainability | \| Risk of declining tool use as clinicians internalise recommendations; interface to patient materials remains key to maintain added value. Impact may persist if recommendations are applied despite reduced use. \| \| --- \| | Reinforce patient-material interface and consultation integration  Probably tool use may decline over time while impact (more appropriate imaging) is maintained |

Table 2 Supplement 5 Planned implementation strategy components mapped to the ERIC taxonomy for dissemination and integration of Betti in German primary care

| **ERIC implementation strategy** | **Operationalisation in Betti** |
| --- | --- |
| Access new funding | Preparation of an implementation-focused funding proposal |
| Build a coalition  Develop academic partnerships | Collaboration with other teams developing comparable complex interventions to coordinate implementation efforts and share infrastructure/learning |
| Change infrastructure | Strengthen in-consultation integration (e.g., streamlined workflow, direct handover to patient materials, practice software integration) to improve sustainability and patient access |
| Conduct educational meetings  Conduct ongoing training  Create a learning collaborative  Distribute educational materials  Promote network weaving | Dissemination through established clinical networks (e.g., clinical information platforms; professional training networks)  CME-accredited sessions (e.g., quality circles, courses, conferences)   \| public symposia (online/in-person) for broader audience  Placement and distribution of educational material via professional journals/newsletters, clinical platforms, and printed materials in care/community settings (posters/flyers in GP practices, physiotherapy, pharmacies; potentially occupational medicine \| \| --- \| |
| Develop a formal implementation blueprint | A structured implementation plan with roles, timelines, delivery pathways, and evaluation indicators, to be finalised in the future implementation study |
| Develop and implement tools for quality monitoring | Monitoring of key delivery indicators (e.g., training completion, usage signals, handover to patient materials) to support ongoing improvement. |
| Obtain and use patients/consumers and family feedback | Structured feedback channel via the intervention website (e.g., comment function) to inform iterative improvements |
| Develop educational materials | Multimodal materials for clinicians and patients (text, video, podcast), iteratively refined using expert and practice-test feedback. |
| Identify early adopters | Targeting GP trainees and training contexts as an entry point for early uptake and diffusion. |
| Inform local opinion leaders | Engagement of educators and trainers involved in GP training to support credibility and dissemination. |
| Make billing easier | Exploration of reimbursement pathways (e.g., new billable care model) and contact with health insurers to support delivery and reduce financial barriers |
| Tailor strategies | Systematic tailoring based on practice-test findings and expert input (e.g., reducing time burden; improving handover to patient materials; adapting content/format) |
| Use mass media | Patient-facing articles and social media; outreach linked to awareness days (e.g. International Day of Radioglogy, World Patient Safety Day |
